# Ranking procedures for repeated measures designs with missing data: Estimation, testing and asymptotic theory

**DOI:** 10.1177/09622802211046389

**Published:** 2021-11-29

**Authors:** Kerstin Rubarth, Markus Pauly, Frank Konietschke

**Affiliations:** 1 14903 Charité - Universitätsmedizin Berlin, Corporate Member of Freie Universität Berlin and Humboldt-Universität zu Berlin, Institute of Biometry and Clinical Epidemiology, Charitéplatz 1, Berlin, Germany; 2 Berlin Institute of Health (BIH), Anna-Louisa-Karsch-Straße 2, Berlin, Germany; 3 Department of Statistics, TU Dortmund University, Dortmund, Germany

**Keywords:** Rank statistics, nonparametric methods, relative effect, repeated measurements, missing data

## Abstract

We develop purely nonparametric methods for the analysis of repeated measures designs with missing values. Hypotheses are formulated in terms of purely nonparametric treatment effects. In particular, data can have different shapes even under the null hypothesis and therefore, a solution to the nonparametric Behrens-Fisher problem in repeated measures designs will be presented. Moreover, global testing and multiple contrast test procedures as well as simultaneous confidence intervals for the treatment effects of interest will be developed. All methods can be applied for the analysis of metric, discrete, ordinal, and even binary data in a unified way. Extensive simulation studies indicate a satisfactory control of the nominal type-I error rate, even for small sample sizes and a high amount of missing data (up to 30%). We apply the newly developed methodology to a real data set, demonstrating its application and interpretation.

## 1 Introduction

Repeated measures (RM) designs are commonly used in various research areas and especially in biomedicine. In such layouts, subjects (e.g. patients) are observed under different time points or experimental conditions allowing for statistical inference within a longitudinal framework. A special example is given by a paired design in which each subject is observed twice. Even though RM designs might be more efficient and a cost saving alternative to general factorial designs involving independent units only, missing values might occur, which aggravate both the statistical modeling and evaluation tremendously. Besides determining the missing value mechanism (missing completely at random (MCAR), missing at random (MAR) or missing not at random), estimation of treatment effects along with testing hypotheses of interest becomes a challenging part. Up to now, many powerful parametric (mean-based) as well as purely nonparametric (rank-based) statistical methods exist for data evaluations. To name a few, RM analysis of variance (RM-ANOVA), linear mixed models, or generalized estimation equations are well-established parametric tools that can be used to analyze RM with missing data (assuming normality and specific covariance matrices). For a detailed overview we refer to Little and Rubin^1^. Brunner et al.^2^ and Domhof et al.^3^ propose purely nonparametric rank-based methods, which do not rely on any distributional assumption and can be used for analyzing metric, discrete or even ordinal data in a unified way. While these ranking methods assume MCAR data, Akritas et al.^4^ propose a generalized approach for bivariate data that is valid under a mixture of MCAR and MAR observations. The methods are known to be powerful and robust (with respect to data distributional shapes) and, in particular, they are invariant under any monotone transformation of the data. Therefore, ranking procedures are often preferred for making statistical inference in ordinal data, in general. In RM designs with missing values, however, the application of existing ranking methods has some disadvantages, which are all motivated from a practical point of view:

1. The procedures can only be used to test global null hypotheses formulated in terms of the distribution functions (i.e. all distributions are identical). Thus, they do not allow for *variance heteroscedasticity* under the null hypothesis. But, allowing for different variances makes the statistical method more flexible and robust to model mis-specifications.
2. Testing the *global null hypothesis* usually does not answer the main research question of the practitioners; inferring linear contrasts in means of the effects of interest to detect local and specific differences is of practical importance (controlling the family wise error rate in the strong sense).
3. The methods cannot be inverted into *confidence intervals* for the treatment effects. Confidence intervals, however, are used to display variability in the data and shall complement any decent statistical analysis. In particular, international regulatory authorities (e.g. international conference on harmonization ICH) require the computation of confidence intervals (see ICH E9 for clinical trials).
4. The procedures proposed by Domhof et al.^3^ might not even be *computable*, because the estimator of the proposed variance-covariance matrix is not necessarily positive semidefinite^3^.

The present paper aims to improve upon these points and thus foster the applicability of nonparametric methods. As a specific example, we propose a solution to the nonparametric Behrens-Fisher problem in RM designs with missing data. All of the proposed methods use all-available data and are valid under the MCAR mechanism. In particular, to tackle point 4, the paper proposes a positive-semidefinite estimator of the variance-covariance matrix of the rank means, that is consistent under arbitrary (but fixed) alternatives. The estimation of the covariance matrix as proposed by Konietschke et al.^5^ cannot be easily transferred to the case of incomplete data. Thus, this estimation problem along with the development of statistical methods is the main focus of this paper. As a side note, we also introduce a new approximation of the distribution of the ANOVA-type statistic of Konietschke et al.^5^ via the Greenhouse-Gaisser method, first introduced by Box^6^. The remainder of the paper is organized as follows. A motivating example with real data is given in section “Motivating example”. In the next section “Nonparametric statistical model and effects”, the statistical model is introduced. Existing ranking methods and their limitations are discussed in section “Existing rank methods and their limitations”. Point estimators along with their asymptotic distributions are exemplified in section “Estimators and their asymptotic distribution” followed by positive semidefinite estimation of the variance covariance matrix in section “Estimation of the covariance matrix”. Test procedures and confidence intervals are provided in Section “Test statistics”. Results of extensive simulation studies are presented in Section “Simulation study”, where we exemplify the behavior of the methods in case of MCAR and MAR scenarios. It turns out that the methods are also applicable in MAR scenarios. The paper closes with the evaluation of the illustrative example in section “Analysis of the example” and a discussion about the findings in section “Discussion and conclusions”. Technical proofs and additional results of the simulation study can be found in the supplementary material.

## 2 Motivating example

**Figure 1.**
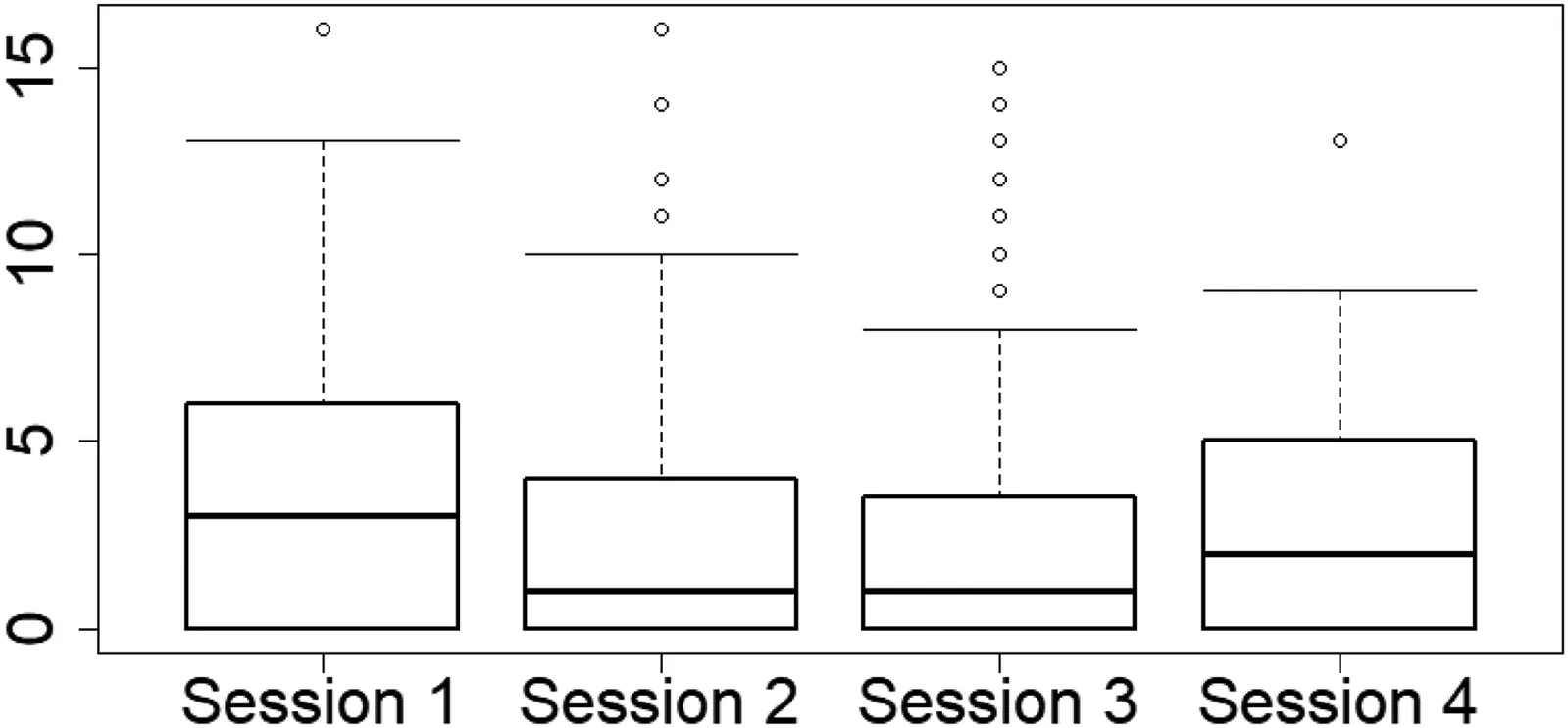
Boxplots of the migraine severity level for all four sessions—all available cases.

As a motivating example we consider a migraine trial data set, which has already been investigated by Kostecki-Dillon et al.^7^, Gao^8^, and Konietschke et al.^9^. In total 135 patients were enrolled in a non-drug headache program, which consisted of four consecutive sessions. In each session, the headache severity level was measured on an ordinal scale ranging from 0 to 20. The lower the score, the better the clinical outcome. The objective of this study is to investigate whether the scores change during the four consecutive sessions. Boxplots of the scores are displayed in Figure 1. It can be readily seen that scores decrease (on median) until session 3 and slightly increase in the last session. However, the data set contains a large amount of missing values, out of the 135 patients, only 33 could be observed in each of the four sessions. Table 1 displays the exact missing pattern. Gao^8^ performed correlation analyses of the missing proportions versus the headache severity level and concluded that assuming MCAR mechanism is reasonable for the study. Note that the data is measured on an ordinal scale and is highly skewed. Therefore, calculating means and applying mean-based inference methods for analyzing this data set is inappropriate. As a remedy, a purely nonparametric statistical model will be introduced in the next section.

**Table 1.** Missing pattern in migraine trial, O = observed, M = missing.

Session 1	Session 2	Session 3	Session 4	Total	%
O	O	O	O	33	24.44
O	O	O	M	48	35.56
O	O	M	M	42	31.11
O	M	M	M	2	1.48
M	O	O	O	2	1.48
M	M	O	O	4	2.96
M	M	M	O	1	0.74
M	M	M	M	2	1.48
O	M	O	O	1	0.74
				135	100

## 3 Nonparametric Statistical Model and Effects

The data example from Section “Motivating example” can be described by n independent and identically distributed d -dimensional random vectors

(1) $$X_{k}=((\lambda_{1k},X_{1k}),\ldots ,(\lambda_{dk},X_{dk}){)}^{\prime },\;k=1,\ldots ,n,\;\mathrm{with}$$

(2) $$\begin{array}{r} \lambda_{ik}=\left\{ \begin{array}{cc} 1, & X_{ik}\;\mathrm{is}\;\mathrm{observed}\\ 0, & X_{ik}\;\mathrm{is}\;\mathrm{missing}, \end{array}\right. \end{array}$$

and marginal distributions $X_{ik}∼F_{i}(x)$ . The indicators $\lambda_{ik}$ are known constants and used for convenient notation. In order to account for metric, discrete, ordered categorical, and even dichotomous data in a unified way, we use the normalized version of the distribution function

(3) $$F_{i}(x)=P(X_{ik}<x)+\frac{1}{2}P(X_{ik}=x),i=1,\ldots ,d;k=1,\ldots ,n,$$

which is the average of the left- and the right continuous versions $F_{i}^{-}(x)=P(X_{i1}<x)$ and $F_{i}^{+}(x)=P(X_{i1}\leq x)$ of the distribution function, respectively. The normalized version of the distribution function was first mentioned by Ruymgaart^10^ and was later used by several authors developing rank statistics in the case of ties. A detailed overview is provided by Brunner et al.^11^.

In model (1), the numbers of non-missing observations under condition i and in total are given by

(4) $$\lambda_{i}=\sum_{k=1}^{n}\lambda_{ik}\;\;\mathrm{and}\;\;N=\sum_{i=1}^{d}\lambda_{i},$$

respectively. In order to derive asymptotic results, we consider the following general framework:

(5) $$\left(A1\right)\;\;\;\lambda_{i}\to \infty ,\;i=1,\ldots ,d,$$

(6) $$\left(A2\right)\;n\to \infty \;\mathrm{such}\;\mathrm{that}\;\frac{n}{\lambda_{i}}\leq N_{0}<\infty ,N_{0}\;\mathrm{being}\;\mathrm{an}\;\mathrm{arbitrary}\;\mathrm{constant}.$$

These assumptions imply that asymptotic results hold even when the numbers of missing values are bounded, which is the most realistic case. Beyond that, other assumptions as, e.g. specific pattern of missing values, are not required. Model (3), however, does not contain any parameters which could be used to define an appropriate treatment effect. To accomplish this, Domhof et al.^3^ propose to use the marginal distributions within the *weighted* *relative marginal effects*

(7) $$r_{i,N}=\int H_{N}dF_{i}=P(Y<X_{i1})+\frac{\frac{1}{2}}{P}(Y=X_{i1})\text{, }i=1,\ldots ,d.$$

Here, $H_{N}=\frac{1}{N}\sum_{i=1}^{d}\lambda_{i}F_{i}(x)$ denotes a *weighted* mean distribution function and $Y∼H_{N}$ . Since $H_{N}$ depends on the amount of missing data, $r_{i,N}$ is not a model constant and cannot be used to quantify causal differences between the distributions a priori. Testing hypotheses formulated in terms of the weighted relative effects as well as computing confidence intervals for them would be dubious. Following Konietschke et al.^12^ and Brunner et al.^13^ for the complete case setting, we therefore propose to use *unweighted* relative effects

(8) $$p_{i}=\int GdF_{i}=P(Z<X_{i1})+\frac{\frac{1}{2}}{P}(Z=X_{i1})\text{, }i=1,\ldots ,d,$$

see also Umlauft et al.^14^. Here, $G=\frac{1}{d}\sum_{s=1}^{d}F_{s}$ denotes the unweighted mean distribution function and Z∼G , independent of $X_{i1}$ . Thus, $p_{i}$ relates the distribution $F_{i}$ *relatively* to the mean distribution G and models whether observations coming from $F_{i}$ tend to result in larger values than those from G . If $p_{i}<p_{j}$ , then data coming from $F_{i}$ tend to be smaller than those coming from $F_{j}$ . If $p_{i}=p_{j}$ , then none of the observations tend to be smaller or larger. *No treatment effect* is therefore indicated as Cp=0 , where C denotes an appropriate contrast matrix and $p=(p_{1},\ldots ,p_{d}{)}^{\prime }$ denotes the vector of all relative marginal effects. Note that the null hypothesis $H_{0}^{F}\;:\;CF=0$ implies $H_{0}^{p}\;:\;Cp=0$ , whereas the reverse does not hold in general, as can be easily seen in normal distribution models. Therefore, testing $H_{0}^{p}$ is known as the nonparametric Behrens-Fisher problem^15^. We note that Domhof^16^ develops univariate confidence intervals for the effects $p_{i}^{*}=\frac{1}{d-1}\sum_{s\neq i}\int F_{s}dF_{i}$ , which, however, might result in paradox conclusions, for example, non-transitive relative effects and we therefore do not follow this approach further. The asymptotic results however, are similar. First, existing rank methods for testing the null hypothesis $H_{0}^{F}\;:\;CF=0$ will be discussed.

## 4 Existing rank methods and their limitations

It has been shown in (7) that the weighted relative marginal effect $r_{i,N}$ is a summary measure of the marginal distribution functions $F_{i}(x)$ and $H_{N}(x)$ . A consistent estimator of $r_{i,N}$ is now obtained by replacing each marginal distribution function $F_{i}(x)$ and $H_{N}(x)$ with their empirical counterpart within the integral representation of $r_{i,N}$ in (7). In order to account for possibly missing values, we define the empirical distribution function of the data under condition i as the average of the all-available data by

(9) $${\hat{F}}_{ik}(x)=\left\{ \begin{array}{cc} c(x-X_{ik}), & \lambda_{ik}=1\\ 0, & \lambda_{ik}=0 \end{array}\right.\;\mathrm{resulting}\;\mathrm{in}\;\;{\hat{F}}_{i}(x)=\frac{1}{\lambda_{i}}\sum_{k=1}^{n}{\hat{F}}_{ik}(x).$$

Here, c(u)=0,1/2,1 , according as u<,=,>0 , denotes the normalized version of the count function. Furthermore, let ${\hat{H}}_{N}(x)=\frac{1}{N}\sum_{i=1}^{d}\lambda_{i}{\hat{F}}_{i}(x)$ denote the empirical counterpart of $H_{N}$ and note that $R_{ik}=\lambda_{ik}(N{\hat{H}}_{N}(X_{ik})+\frac{\frac{1}{2}}{)}$ is the mid-rank of $X_{ik}$ among all N observed values. If $\lambda_{ik}=0$ and thus $X_{ik}$ is missing, $R_{ik}=0$ , for convenience. Plugging-in ${\hat{H}}_{N}$ and ${\hat{F}}_{i}$ into (7) leads to a rank estimator of $r_{i,N}$ as

(10) $${\hat{r}}_{i,N}=\int {\hat{H}}_{N}d{\hat{F}}_{i}=\frac{1}{\lambda_{i}}\sum_{k=1}^{n}\lambda_{ik}{\hat{H}}_{N}(X_{ik})=\frac{1}{N}\left({\bar{R}}_{i\cdot }-\frac{1}{2}\right).$$

Here, ${\bar{R}}_{i\cdot }=\lambda_{i}^{-1}\sum_{k=1}^{n}R_{ik}$ denotes the mean of the ranks under condition i . For convenient representation of asymptotic results, the point estimators are collected in the vector ${\hat{r}}_{N}=({\hat{r}}_{1,N},\ldots ,{\hat{r}}_{d,N}{)}^{\prime }$ .

Akritas and Brunner^17^ have shown that $\sqrt{n}C{\hat{r}}_{N}$ follows, asymptotically, as n→∞ , a multivariate normal distribution with expectation 0 and covariance matrix $CV_{n}C$ under the special null hypothesis $H_{0}^{F}\;:\;CF=0$ , where $V_{n}=Cov(\lambda_{11}H_{N}(X_{11}),\ldots ,\lambda_{d1}H_{N}(X_{d1}){)}^{\prime }$ . The asymptotic distribution of the estimators under (any) alternative has not yet been developed. Moreover, since $V_{n}$ is unknown in practical applications and must be estimated from the data, Domhof et al.^3^ propose to use ${\hat{V}}_{n}=(\hat{v}(i,i^{\prime }){)}_{i,i^{\prime }=1,\ldots ,\;d}$ with

(11) $$\begin{array}{rl} \hat{v}(i,i)= & \frac{n}{N^{2}\lambda_{i}(\lambda_{i}-1)}\sum_{k=1}^{n}\lambda_{ik}(R_{ik}-{\bar{R}}_{i.}{)}^{2},\\ \hat{v}(i,i^{\prime })= & \frac{n}{NK(i,i^{\prime })}\sum_{k=1}^{n}\lambda_{ik}\lambda_{i^{\prime }k}(R_{ik}-{\bar{R}}_{i.})(R_{i^{\prime }k}-{\bar{R}}_{i^{\prime }.}). \end{array}$$

Here $K(i,i^{\prime })=(\lambda_{i}-1)(\lambda_{i^{\prime }}-1)+\Lambda (i,i^{\prime })-1$ with $\Lambda (i,i^{\prime })=\sum_{k=1}^{n}\lambda_{ik}\lambda_{i^{\prime }k}$ , see Brunner et al.^18^ and Domhof et al.^3^. The estimator ${\hat{V}}_{n}$ , however, is not necessarily positive semidefinite in case of missing values and thus might result in a negative variance estimator of a linear combination of the estimators ${\hat{r}}_{i,N}$ . In addition, since the distribution of the estimators is only known under the null hypothesis $H_{0}^{F}$ , confidence intervals for the effects of interest—even without missing values—cannot be computed. In the next section, consistent estimators of the unweighted relative effects $p_{i}$ along with a positive semidefinite estimator of its variance-covariance matrix will be proposed. The estimator is even consistent under general alternatives formulated in terms of p .

## 5 Estimators and their asymptotic distribution

Following the above ideas, a point estimator for $p_{i}$ in (8) is readily available by replacing each of the unknown distribution functions $F_{i}(x)$ and G(x) with their empirical counterparts ${\hat{F}}_{i}(x)$ in the integral representation of $p_{i}$ . Plugging-in the empirical counterpart

$$\hat{G}(x)=\frac{1}{d}\sum_{s=1}^{d}{\hat{F}}_{s}(x)$$

of G(x) in (8) leads to the point esimator

(12) $${\hat{\;p}}_{i}=\int \hat{G}d{\hat{F}}_{i}=\frac{1}{\lambda_{i}}\sum_{k=1}^{n}\frac{\lambda_{ik}}{d}\sum_{s=1}^{d}\frac{1}{\lambda_{s}}\sum_{\ell =1}^{n}\lambda_{s\ell }c(X_{ik}-X_{s\ell }).$$

In general, the estimator cannot be computed using ranks of the data; instead it is a sum of indicators (values of the count functions) and therefore its numerical computation is slightly more involved than that of ${\hat{r}}_{i,N}$ . If no missing values are apparent, then ${\hat{r}}_{i,N}={\hat{\;p}}_{i},i=1,\ldots ,d$ . First its asymptotic properties will be studied in the following proposition.

Proposition 1. The estimator $\hat{p}=({\hat{\;p}}_{1},\ldots ,{\hat{\;p}}_{d}{)}^{\prime }$ is asymptotically unbiased and strongly consistent under **(A1)** in (5), i.e.

1.
$E(\hat{p})=p+O(\frac{1}{n})$

2.
$\hat{p}-p\to^{a.s.}0,\;\min\{\lambda_{1},\ldots ,\lambda_{d}\}\to \infty$ .

Next, the asymptotic distribution of the statistic $\sqrt{n}(\hat{p}-p)$ will be derived. The following Theorem 1 shows that $\sqrt{n}(\hat{p}-p)$ has, asymptotically, under **(A1)** and **(A2)**, the same distribution as the random vector $\sqrt{n}B=\sqrt{n}(B_{1},\ldots ,B_{d}{)}^{\prime }$ , whose components are sums of independent random variables:

(13) $$\begin{array}{rl} \sqrt{n}B_{i}= & \frac{1}{\sqrt{n}}\sum_{k=1}^{n}\left(\Psi_{ik}-E\left(\Psi_{ik}\right)\right),\;\mathrm{where},\\ \Psi_{ik}= & \frac{n\lambda_{ik}}{\lambda_{i}}\left(G(X_{ik})-\frac{1}{d}F_{i}(X_{ik})\right)-\frac{1}{d}\sum_{s\neq i}\frac{n\lambda_{sk}}{\lambda_{s}}F_{i}(X_{sk}),\;\mathrm{and}\\ E\left(\Psi_{ik}\right)= & \frac{n\lambda_{ik}}{\lambda_{i}}\left(\;p_{i}-\frac{1}{d}p^{\left(ii\right)}\right)-\frac{1}{d}\sum_{s\neq i}\frac{n\lambda_{sk}}{\lambda_{s}}p^{\left(is\right)}. \end{array}$$

Here, $p^{\left(is\right)}=\int F_{i}dF_{s}$ and $p^{\left(ii\right)}=\int F_{i}dF_{i}=\frac{1}{2}$ denote pairwise defined relative marginal effects between time points i and s and i and i , respectively.

Theorem 1.
Let $\sqrt{n}B=\sqrt{n}(B_{1},\ldots ,B_{d}{)}^{\prime }$ be the vector of the random variables $\sqrt{n}B_{i},i=1,\ldots ,d$ , as defined in (13). If n→∞ such that **(A1)** and **(A2)** in (5) and (6) hold, then,

$${\left|\left|\sqrt{n}\left(\hat{p}-p\right)-\sqrt{n}B\right|\right|}_{2}^{2}=O\left(\frac{1}{n}\right),$$

with $\|x{\|}_{2}^{2}$ denoting the $L_{2}$ -norm.

It follows from Theorem 1, that the asymptotic covariance matrix of the linear rank statistic $\sqrt{n}(\hat{p}-p)$ is given by

(14) $$V_{n}=Cov(\sqrt{n}B).$$

The asymptotic multivariate normality of the linear rank statistic $\sqrt{n}(\hat{p}-p)$ is given in the next Theorem.

Theorem 2.
Under the assumptions **(A1)** and **(A2)**, the statistic $\sqrt{n}(\hat{p}-p)$ follows asymptotically, as n→∞ , a multivariate normal distribution with expectation 0 and covariance matrix $V_{n}$ .

The covariance matrix $V_{n}$ , however, is unknown in practical applications and must be estimated from the data for making statistical inferences. We will derive a consistent and positive-semidefinite estimator in the next section.

## 6 Estimation of the covariance matrix

If the random variables $\Psi_{ik}$ in (13) were observable, then an estimator of $V_{n}$ would be given by

$${\tilde{V}}_{n}=\frac{1}{n-1}\sum_{k=1}^{n}\left(\Psi_{k}-E(\Psi_{k})\right){\left(\Psi_{k}-E(\Psi_{k})\right)}^{\prime }.$$

Note that in the definition of ${\tilde{V}}_{N}$ each vector $\Psi_{k}=(\Psi_{1k},\ldots ,\Psi_{dk}{)}^{\prime }$ is centered with its own specific expectation and therefore, ${\tilde{V}}_{n}$ is not the empirical covariance matrix of the vectors $\Psi_{k}$ . However, the variables $\Psi_{k}$ are not observable and thus, ${\tilde{V}}_{n}$ cannot be computed in practical applications. Therefore, we replace them with observable random variables, which are ‘close enough’ to $\Psi_{k}$ in an appropriate norm. Define the vectors ${\hat{\Psi }}_{k}=({\hat{\Psi }}_{1k},\ldots ,{\hat{\Psi }}_{dk}{)}^{\prime }$ , the components of which are the empirical counterparts

$$\begin{array}{rl} {\hat{\Psi }}_{ik}= & \frac{n\lambda_{ik}}{\lambda_{i}}\left(\hat{G}(X_{ik})-\frac{1}{d}{\hat{F}}_{i}(X_{ik})\right)-\frac{1}{d}\sum_{s\neq i}\frac{n\lambda_{sk}}{\lambda_{s}}{\hat{F}}_{i}(X_{sk}),\;\mathrm{and}\\ {\hat{\beta }}_{ik}= & \frac{n\lambda_{ik}}{\lambda_{i}}\left({\hat{\;p}}_{i}-\frac{1}{d}{\hat{\;p}}^{\left(ii\right)}\right)-\frac{1}{d}\sum_{s\neq i}\frac{n\lambda_{sk}}{\lambda_{s}}{\hat{\;p}}^{\left(is\right)}, \end{array}$$

with ${\hat{\;p}}^{\left(is\right)}=\int {\hat{F}}_{i}d{\hat{F}}_{s}$ and ${\hat{\;p}}^{\left(ii\right)}=\int {\hat{F}}_{i}d{\hat{F}}_{i}=\frac{1}{2}$ . Furthermore, consider the estimator

(15) $${\hat{V}}_{n}=\frac{1}{n-1}\sum_{k=1}^{n}\left({\hat{\Psi }}_{k}-{\hat{\beta }}_{k}\right){\left({\hat{\Psi }}_{k}-{\hat{\beta }}_{k}\right)}^{\prime },\;{\hat{\beta }}_{k}=({\hat{\beta }}_{1k},\ldots ,{\hat{\beta }}_{dk}{)}^{\prime }.$$

Its properties are listed in the next Theorem.

Theorem 3.
Let $V_{n}=Cov(\sqrt{n}B)$ and let ${\hat{V}}_{n}$ as given in (15). Then,

1.
${\hat{V}}_{n}$ is positive semidefinite.
2. If n→∞ such that **(A1)** and **(A2)** hold, then ${\hat{V}}_{n}-V_{n}\to^{a.s.}0$ .

## 7 Test statistics

In this section, we introduce different test procedures (global and multiple) to test the null hypothesis $H_{0}^{p}\;:\;Cp=0$ . First, quadratic test procedures will be explained and a linear multiple contrast test procedure (MCTP) will be introduced afterwards. One advantage of the MCTP over the quadratic test procedures is, that it can be used for testing multiple hypotheses as well as for constructing simultaneous confidence intervals for the relative marginal effects $p_{i}$ and linear combinations thereof. In particular, the adjusted *p*-values and the corresponding confidence intervals are compatible, that is, it is not possible that the null hypothesis is rejected by the multiple comparison procedure, but the corresponding confidence interval includes $\frac{1}{2}$ - the hypothetical value of no treatment under $H_{0}^{p}$ .

### 7.1 Quadratic tests

Domhof et al.^3^ introduce two different types of test statistics to infer the global null hypothesis $H_{0}^{F}\;:\;CF=0$ formulated in terms of the distribution functions. Since both of the methods only depend on the vector of point estimators, their estimated variance-covariance and hypothesis (contrast) matrices, they can be generalized to test $H_{0}^{p}\;:\;Cp=0$ using the newly developed estimators. Consider the Wald-type statistic (WTS)

(16) $$Q_{n}=n{\hat{p}}^{\prime }C^{\prime }[C{\hat{V}}_{n}C^{\prime }{]}^{+}C\hat{p},$$

which is a quadratic form in the point estimators $\hat{p}$ . Under $H_{0}^{p}$ , the distribution of $Q_{n}$ can be approximated by a $\chi_{\hat{f}}^{2}$ distribution with $\hat{\;f}=rank(C{\hat{V}}_{n}C^{\prime })$ degrees of freedom. Here, $[\cdot {]}^{+}$ denotes the Moore-Penrose inverse of a matrix. Simulation studies indicate, however, that the test upon $Q_{n}$ behaves liberal and highly over-rejects the null hypothesis when sample sizes are small. Therefore, Domhof et al.^3^ propose to approximate its distribution by an F-distribution resulting in the so-called ANOVA-type statistic. Let $M=C^{\prime }[CC^{\prime }{]}^{-}C$ be a projection matrix and let $[CC^{\prime }{]}^{-}$ be a generalized inverse of $CC^{\prime }$ . Then, the null hypotheses $H_{0}^{p}(C)\;:\;Cp=0$ and $H_{0}^{p}(M)\;:\;Mp=0$ are equivalent. It holds under $H_{0}^{p}(M)$ that the distribution of the ATS

(17) $$A_{n}=\frac{ntr(M{\hat{V}}_{n})}{tr(M{\hat{V}}_{n}M{\hat{V}}_{n})}{\hat{p}}^{\prime }M\hat{p}$$

can be approximated by a $\chi_{\hat{\;f}}^{2}$ distribution with

(18) $$\hat{\;f}=\frac{[tr(M{\hat{V}}_{n}){]}^{2}}{tr(M{\hat{V}}_{n}M{\hat{V}}_{n})}$$

degrees of freedom. Here, tr(A) denotes the trace of the matrix A . However, simulation studies indicate a liberal behavior of the ATS in some settings^5^. We therefore propose to apply the Greenhouse-Gaisser method introduced by Box^6^ resulting in

(19) $$A_{n,2}=\frac{n{\hat{p}}^{\prime }M\hat{p}}{tr(M{\hat{V}}_{n})}$$

which can be approximated by an $F_{\hat{\;f_{1}},{\hat{\;f}}_{2}}$ distribution with

(20) $${\hat{\;f}}_{1}=\hat{\;f}\;\mathrm{and}\;{\hat{\;f}}_{2}=(n-1)\hat{\;f}$$

degrees of freedom. Note that we will refer to (17) as the first version of the ATS or ATS (1) and to (19) as the second version of the ATS or ATS (2) for convenience. Next, MCTPs along with simultaneous confidence intervals will be introduced.

### 7.2 Multiple contrast test procedure

As already outlined above, the quadratic test procedures can only be used to test the global null hypothesis of no effect. Therefore, Konietschke et al.^5^ propose a MCTP for making local statistical inference. In order to test the individual null hypothesis $H_{0}^{\left(\ell \right)}\;:\;c_{\ell }^{\prime }p=0$ , consider the test statistic

$$T_{\ell }=\sqrt{n}\frac{c_{\ell }^{\prime }(\hat{p}-p)}{\sqrt{c_{\ell }^{\prime }{\hat{V}}_{n}c_{\ell }}}.$$

Here, $c_{\ell }^{\prime }$ denotes the ℓ th row vector of C . Even though the exact distribution of $T_{\ell }$ for finite sample size n remains unknown, it is asymptotically standard normal. Furthermore, the test statistics $T_{\ell }$ and $T_{m}$ are not necessarily independent due to the chosen contrasts $c_{\ell }^{\prime }$ and $c_{m}^{\prime }$ ( ℓ≠m ) and/or the RM data. In order to take the correlations across the q test statistics within the multiplicity adjustment into account, we collect them in the vector

$$T={\left(T_{1},\ldots ,T_{q}\right)}^{\prime }.$$

It follows from Theorem 2 and Slutzky’s Theorem, that T follows, asymptotically, a multivariate normal distribution with expectation 0 and correlation matrix

$$R=D^{-1/2}CV_{n}C^{\prime }D^{-1/2},$$

where D is a diagonal matrix of the diagonal elements of $CV_{n}C^{\prime }$ . For large sample sizes, the individual null hypothesis $H_{0}^{\left(\ell \right)}\;:\;c_{\ell }^{\prime }p=0$ will be rejected at multiple level α , that is, the procedure controls the familywise type-I error rate, if

(21) $$|T_{\ell }|\geq z_{1-\alpha ,2,R},$$

where $z_{1-\alpha ,2,R}$ denotes the two-sided (1−α) -equicoordinate quantile of the N(0,R) distribution^12^. The two-sided (1−α) -equicoordinate quantile satisfies the condition $P(|T_{1}|<z_{1-\alpha ,2,R},\ldots ,|T_{q}|<z_{1-\alpha ,2,R})=1-\alpha$ for $\left(T_{1},\ldots ,T_{q}{)}^{\prime }∼N(0,R\right)$ . Compatible (1−α) -simultaneous confidence intervals for the effects $\delta_{\ell }=c_{\ell }^{\prime }p$ are given by

(22) $$CI_{\ell }=\left[c_{\ell }^{\prime }\hat{p}\mp \frac{z_{1-\alpha ,2,R}}{\sqrt{n}}\sqrt{c_{\ell }^{\prime }{\hat{V}}_{n}c_{\ell }}\;\right].$$

We refer to Bretz et al.^19^ for the numerical derivation of the equicoordinate quantile. Finally, for large sample sizes, the global null hypothesis $H_{0}^{p}\;:\;Cp=0$ will be rejected at two-sided multiple level α , if

(23) $$T_{0}=\max\left\{|T_{1}|,\ldots ,|T_{q}|\right\}\geq z_{1-\alpha ,2,R}.$$

The correlation matrix, however, is unknown in practical applications. We recommend to replace R with its consistent estimator

$${\hat{R}}_{n}={\hat{D}}^{-1/2}C{\hat{V}}_{n}C^{\prime }{\hat{D}}^{-1/2},$$

in (21), (22), and (23), respectively. Here, $\hat{D}$ denotes the diagonal matrix obtained from the diagonal elements of $C{\hat{V}}_{n}C$ . For small sample sizes, we follow Konietschke et al.^5^, who proposed in the case of complete observations to approximate the distribution of T by a multivariate central $t_{n-1}(0,{\hat{R}}_{n})$ distribution. It follows from sections “Estimators and their asymptotic distribution” and “Estimation of the covariance matrix” that both choices lead to asymptotic correct multiple contrast tests.

## 8 Simulation study

All of the procedures developed in the previous sections are of asymptotic nature and thus, investigating their finite sample behavior with respect to their control of the type-I error rate (at nominal 5% level) and power to detect alternatives within extensive simulation studies is mandatory. We considered all introduced tests, namely

1. the WTS in (16) with a critical value from a $\chi_{\hat{\;f}}^{2}$ distribution,
2. the ATS (1) in (17) with the proposed F -approximation,
3. the ATS (2) in (19) with the proposed F -approximation,
4. the MCTP $T_{0}$ in (23) with a $t_{n-1}(0,{\hat{R}}_{n})$ approximation,

and compared them with

1. the WTS and ATS for testing $H_{0}^{F}$ as proposed by Domhof et al.^3^

in different homo- and heteroscedastic RM designs with different rates of missing values. Even though Domhof et al.^3^ reported a liberal behavior of the WTS (for testing $H_{0}^{F}$ ), we added the method as a competing procedure for completeness. We thus also investigated their robustness to variance heteroscedasticity. Since all of the methods above use all-available data, we additionally compared them with two MCTP-based approaches: a complete case analysis and a naive imputation approach, in which we either

1. deleted the whole observation vector $X_{k}$ of subject k if any $X_{ik}$ was missing ( $\lambda_{ik}=0$ ), or
2. if $X_{ik}$ was missing ( $\lambda_{ik}=0$ ), we calculated $median(\lambda_{i1}X_{i1},\ldots ,\lambda_{in}X_{in})$ , and assigned it to $X_{ik}$ and set $\lambda_{ik}=1$ .

Data have been generated using discretized, by rounding to integers, normal and log-normal distributions with varying numbers of time points d∈{3,4} , sample sizes n∈{20,30,50} , amount of missing values r∈{0%,10%,30%} , and six different types of covariance matrices

(24) $$\begin{array}{rl} \Sigma_{1}= & \left(\begin{array}{ccc} 1 & 0.5 & 0.5\\ 0.5 & 1 & 0.5\\ 0.5 & 0.5 & 1 \end{array}\right),\;\;\Sigma_{2}=\left(\begin{array}{cccc} 1 & 0.5 & 0.5 & 0.5\\ 0.5 & 1 & 0.5 & 0.5\\ 0.5 & 0.5 & 1 & 0.5\\ 0.5 & 0.5 & 0.5 & 1 \end{array}\right),\\ \Sigma_{3}= & \left(\begin{array}{ccc} 1 & 0.3 & 0.6\\ 0.3 & 1.2 & 0.9\\ 0.6 & 0.9 & 1.5 \end{array}\right),\;\;\Sigma_{4}=\left(\begin{array}{cccc} 1 & 0.2 & 0.4 & 0.6\\ 0.2 & 2 & 0.7 & 0.5\\ 0.4 & 0.7 & 2.5 & 0.6\\ 0.6 & 0.5 & 0.6 & 3 \end{array}\right),\\ \Sigma_{5}= & \left(\begin{array}{cccc} 1 & 0.6 & 0.36 & 0.216\\ 0.6 & 1 & 0.6 & 0.36\\ 0.36 & 0.6 & 1 & 0.6\\ 0.216 & 0.36 & 0.6 & 1 \end{array}\right),\;\;\Sigma_{6}=\left(\begin{array}{cccc} 1 & 0.8 & 0.64 & 0.512\\ 0.8 & 1.5 & 0.8 & 0.64\\ 0.64 & 0.8 & 2 & 0.8\\ 0.512 & 0.64 & 0.8 & 2.5 \end{array}\right). \end{array}$$

The covariance matrices were chosen to model a broad selection of dependency patterns, including homoscedastic ( $\Sigma_{1}$ and $\Sigma_{2}$ ) as well as heteroscedastic marginals. Note that $H_{0}^{F}$ holds only under $\Sigma_{1}$ and $\Sigma_{2}$ . We furthermore investigated the methods’ sensitivity to both MCAR and MAR data to cover realistic scenarios. In order to generate the former, we multiplied the observations with randomly chosen indicators $\lambda_{ik}∼B(1-r)$ , with a zero entry being interpreted as a missing observation, whereas we followed Santos et. al.^20^ for the latter. Hereby we defined pairs of observations $\left\{X_{obs},X_{miss}\right\}$ , where $X_{obs}$ determines the probability that $X_{miss}$ was actually observed. For instance, in case of d=4 we defined the pairs $\left\{X_{1k},X_{2k}\right\}$ and $\left\{X_{3k},X_{4k}\right\}$ . Following the idea of Amro et al.^21^, we investigated two different types of MAR scenarios, MAR (1) and MAR (2). First, for the MAR (1) scenario, we divided $X_{i,obs}$ into three groups: (1) $\left\{X_{ik}=X_{i,obs}\in (-\infty ,-\sigma_{i}),k=1,\ldots ,n\right\}$ , (2) $\left\{X_{ik}=X_{i,obs}\in (-\sigma_{i},\sigma_{i}),k=1,\ldots ,n\right\}$ , and (3) $\left\{X_{ik}=X_{i,obs}\in (\sigma_{i},\infty ),k=1,\ldots ,n\right\}$ , where $\sigma_{i}^{2}$ is the variance of $X_{i,obs}$ . Then, we assigned a missing rate of 10% to the first and third group and a missing rate of 30% to the second group. Second, in the MAR (2) scenario, data was divided into two groups using the median, following the idea of Zhu et al^22^. Specifically, we defined (1) $\left\{X_{ik}=X_{i,obs}\in (-\infty ,median(X_{i,obs}),k=1,\ldots ,n\right\}$ and (2) $\left\{X_{ik}=X_{i,obs}\in (median(X_{i,obs}),\infty ),k=1,\ldots ,n\right\}$ . Here, we assigned a missing rate of 0% to the first group and a missing rate of 10% to the second group.

For each design, 10,000 simulation runs were performed using the R software package of statistical computing, version R 3.6.4^23^. The complete simulation code is available on https://github.com/KerstinRubarth/RM_Miss.

First, we will discuss simulation results when $H_{0}^{F}$ holds and data is MCAR (Table S. 1). It appears that the ATS for testing $H_{0}^{F}$ tends to be slightly liberal when samples are rather small ( n=20 ). Otherwise, it exhibits an almost accurate type-I error control. In fact, the amount of missing values impact its behavior only by slightly increasing or decreasing the type-I error rate. A similar behavior of the newly developed ATS (1) for testing $H_{0}^{p}$ can be detected. The ATS (2) for testing $H_{0}^{p}$ controls the type-I error even more accurate while sometimes being slightly conservative. Moreover, the new MCTP tends to be quite accurate in most settings and gets slightly liberal if the probability of missingness increases. Contrary, both WTS methods are too liberal for all settings and cannot be recommended. Overall, the simulations for $H_{0}^{F}$ indicate that both versions of the ATS and the MCTP for testing $H_{0}^{p}$ control the type-I error rate quite accurately when n≥20 .

**Figure 2.**
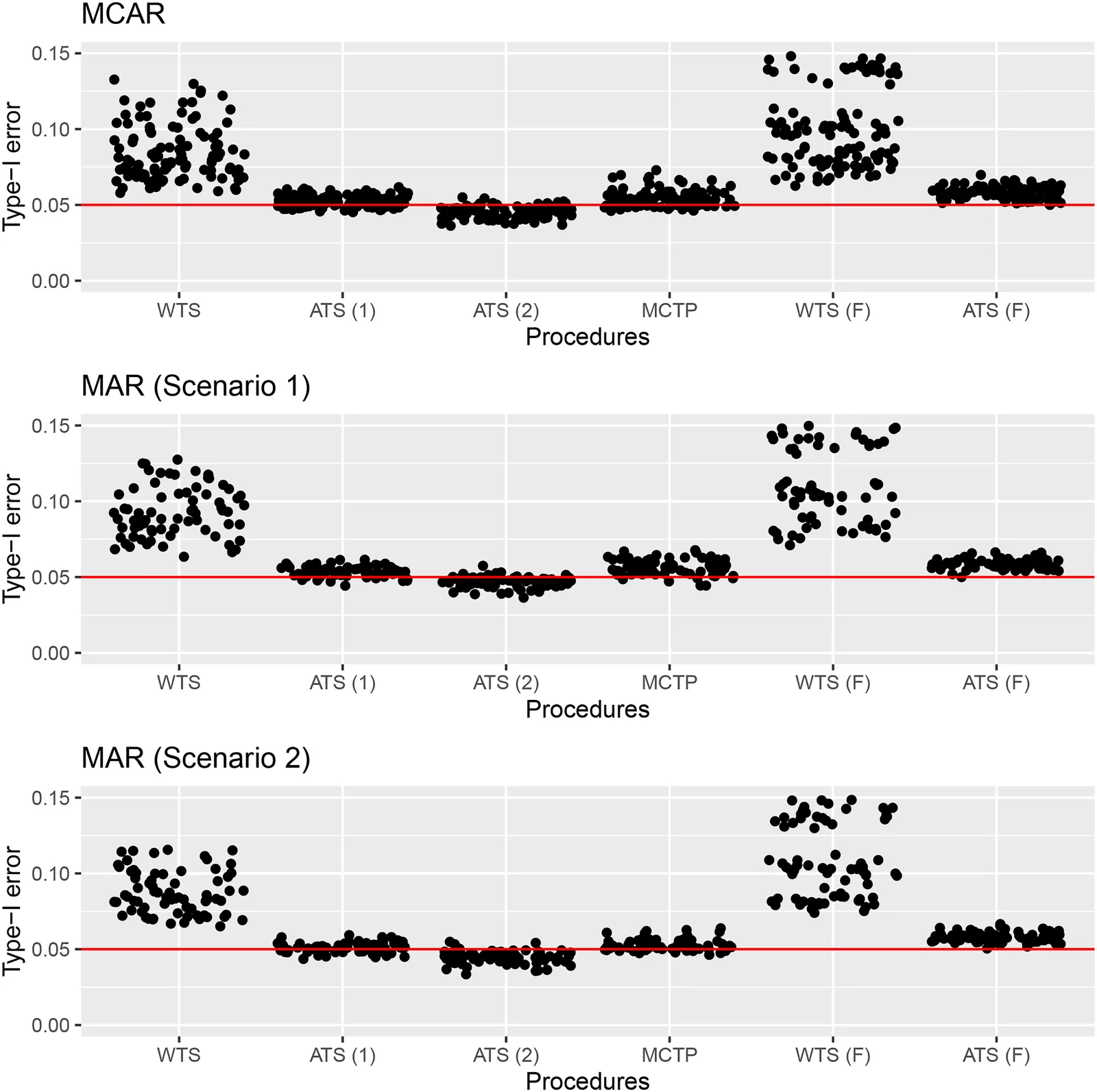
Type-I error rates of the newly proposed Wald- (WTS), ANOVA-type (ATS (1) and ATS (2)) and MCT procedures and the procedures of Domhof et al.^3^ under the three different missing mechanisms MCAR, MAR (1) and MAR (2). MCT: multiple contrast test; MCAR: missing completely at random; MAR: missing at random.

Next, we will explore the methods’ behavior under variance heteroscedasticity (Table S. 2 and Table S. 3). It turns out that the methods tend to be quite accurate in most scenarios and tend to over-reject the null hypothesis when samples are small and missing probabilities are high. However, some scenarios also indicate a fairly robust behavior of the methods for testing $H_{0}^{F}$ . Overall, the methods seem not be too sensitive towards variance heteroscedasticity. In general, the procedures work equally well in case of normally and log-normally distributed data. However, in most scenarios, both versions of the ATS for testing $H_{0}^{p}$ control the type-I error more accurate than the ATS for testing $H_{0}^{F}$ . As already pointed out, both WTS procedures do not control the type-I-error rate in case of smaller sample sizes. Again, for small sample sizes, e.g. n=20 , the ATS (2) under $H_{0}^{p}$ performs better than the MCTP. However, all test statistics show a conservative behavior in case of high correlations and small sample sizes. This was already mentioned in Konietschke et al.^5^ and Friedrich et al.^24^ for the RM design without missing data and Munzel^25^ as well as Harrar et al.^26^ and Amro et al.^27^ for the paired two sample case. Overall, the simulation studies indicate that the newly developed methods based upon the ATS, especially the second version, and (to some extend) the MCTP control the type-I error well when n≥20 . Next, we compare the results of the type-I error simulation for data under MCAR and MAR mechanisms. A graphical overview of the type-I error rates of the procedures for testing $H_{0}^{F}$ and $H_{0}^{p}$ in various settings can be found in Figure 2. Since the missing rates in the MCAR and MAR scenarios in these simulations were similiar and no difference in terms of the type-I error rates is apparent, the procedures seem to be fairly robust to the missing value mechanism, though the theory was developed under the MCAR assumption.

**Figure 3.**
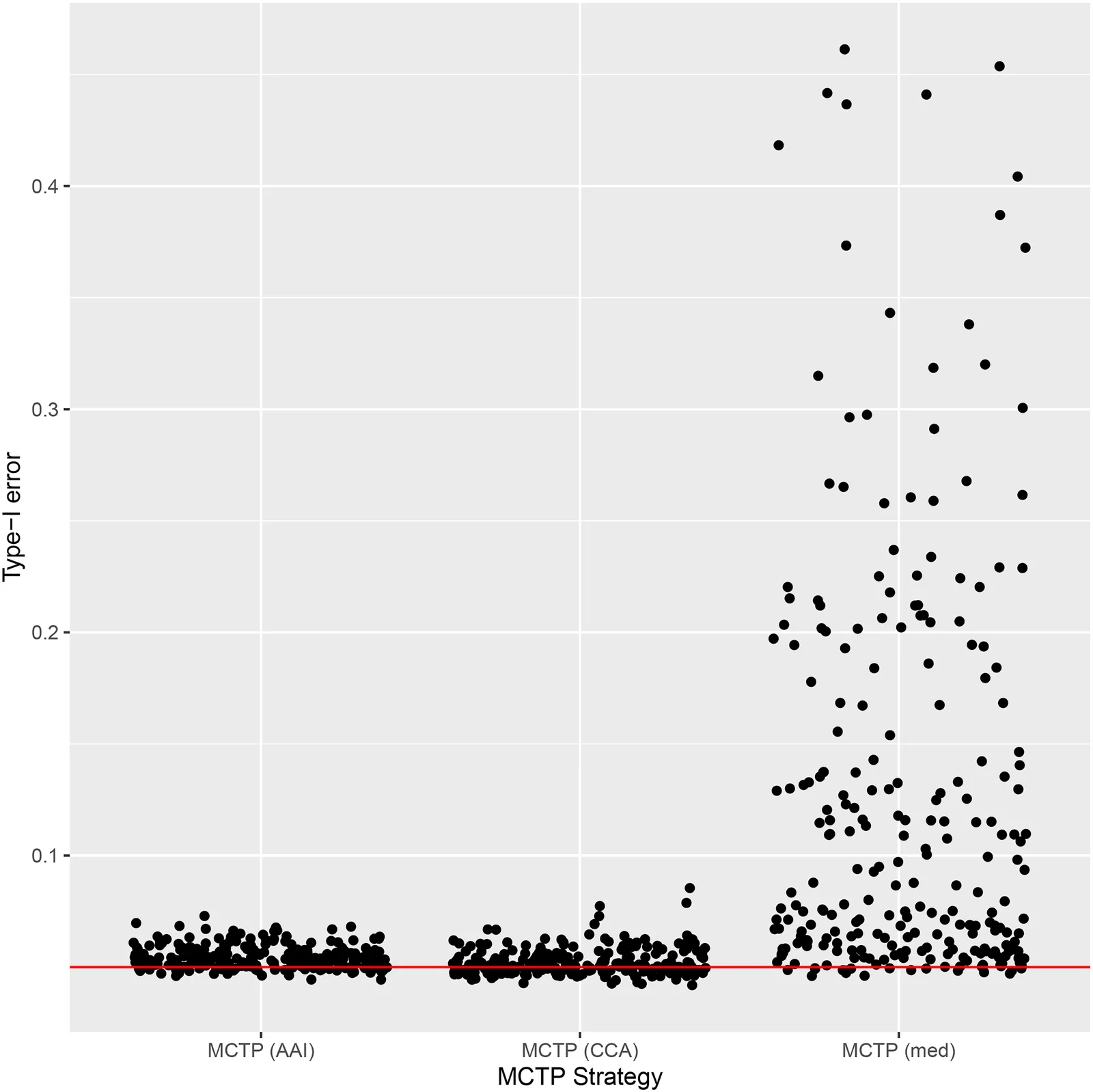
Type-I error rates of the MCTP, either using AAI, only complete cases and an imputed data set, using the median for each repeated measurement. MCTP: multiple contrast test procedure; AAI: all-available information; CCA: Complete Case Analysis; med: median.

Since we advocate to use the MCTP as it additionally allows the simultaneous testing of the corresponding single contrast hypotheses, we want to compare its behavior in terms of type-I-error rates in comparison to two “naive” procedures for handling missing data: median imputation and complete case analysis. A graphical presentation of the results can be found in Figure 3. The results for the newly proposed MCTP which uses all-available information and the MCTP using only complete cases are comparable. As expected (van Buuren^28^, Ramosaj et al.^29^), the simple median imputation yields in some scenarios with many missings extremly inflated type-I error rates and is therefore not recommend. Note that data has been generated under MCAR and MAR assumptions for this comparison.

**Figure 4.**
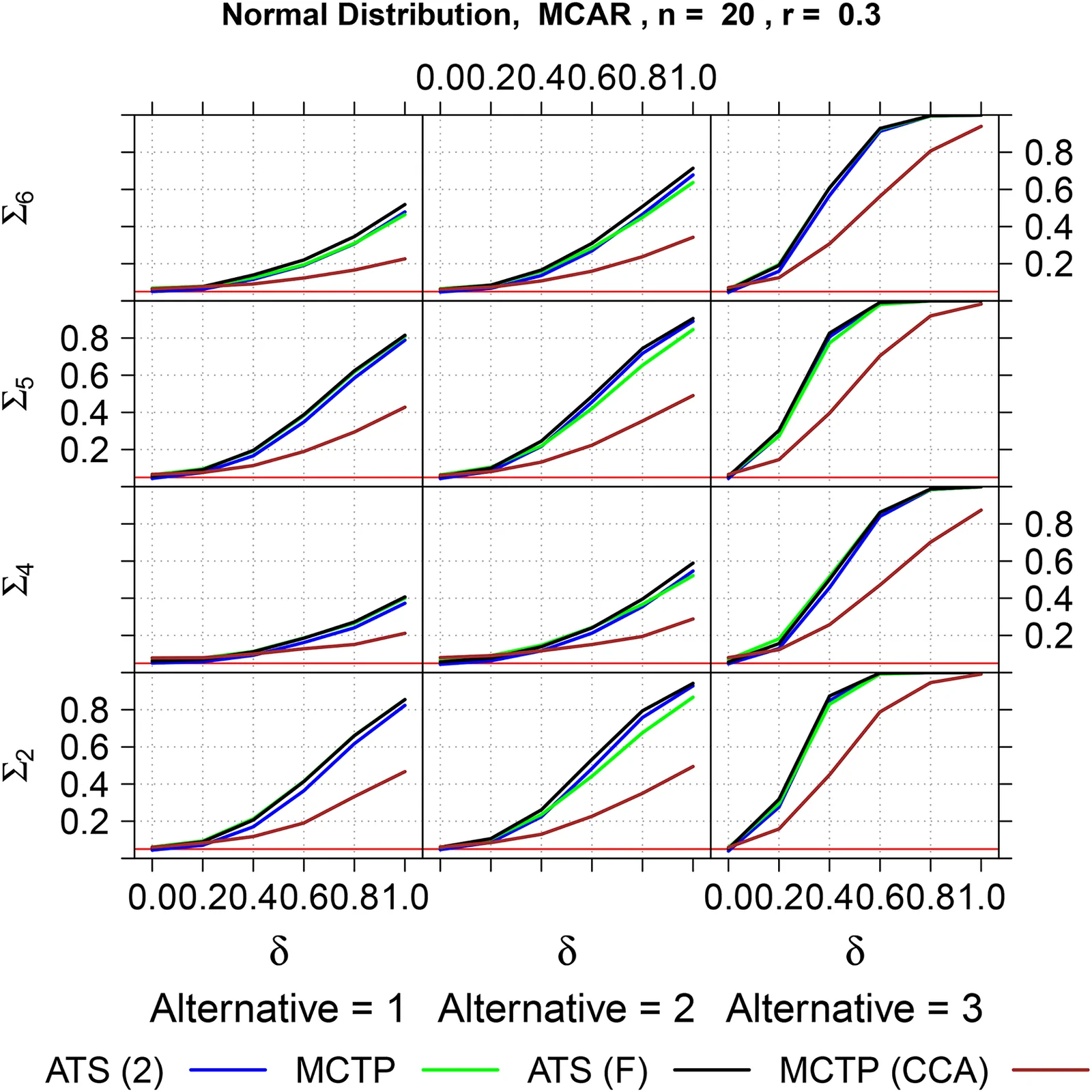
Power simulation of the second version of the ATS and the MCTP for testing $H_{0}^{p}$ , the ATS for testing $H_{0}^{F}$ and the MCTP for testing $H_{0}^{p}$ using only complete cases, data is MCAR. MCTP: multiple contrast test procedure; MCAR: missing completely at random; ATS: ANOVA-type statistic.

In order to investigate the power of the procedures, a simulation study was conducted using four-dimensional normal and log-normal distributions with $\mu =(\mu_{1},\mu_{2},\mu_{3},\mu_{4}{)}^{\prime }$ and covariance matrices $\Sigma_{2},\Sigma_{4},\Sigma_{5},\Sigma_{6}$ . In particular, three different types of shift-alternatives were considered

(25) $$\begin{array}{rll} & Alternative\;1 & Alternative2Alternative3\\ & \mu =(0,0,0,\delta {)}^{\prime }\\ & Alternative\;2\\ & \mu =(0,0,\delta ,\delta {)}^{\prime }\\ & Alternative\;3\\ & \mu =(0,1\delta ,2\delta ,3\delta {)}^{\prime }, \end{array}$$

with ranging δ=(0.2,0.4,0.6,0.8,1,1.5) and different amount of missing values. As the WTS turned out to be inappropriate for small sample sizes, it was not included into the power analysis. Moreover, since the second version of the ATS for testing $H_{0}^{p}$ showed a more accurate behavior than the first version, we only present results for the second version. The results for covariance matrix $\Sigma_{4}$ and n=30 under the MCAR assumption are displayed in Table S. 4 (normal distribution) and S. 5 (log-normal distribution). A graphical overview for $\Sigma_{2},\Sigma_{4},\Sigma_{5},\Sigma_{6}$ under the MCAR assumption is presented in Figures 4 (normal distribution) and 5 (log-normal distribution) for n=20 and r=0.3 . Additionally, we investigated the power of the MCTP using only complete cases, which has a low power compared to the approaches using all-available information due to the decreased sample size. It follows that none of the procedures which use all-availble information is superior in terms of their powers to detect alternatives. However, the MCTP using all available information, provides more information by local test decisions, simultaneous confidence intervals and adjusted *p*-values and is therefore recommended for practical applications despite beeing slightly liberal in some cases.

**Figure 5.**
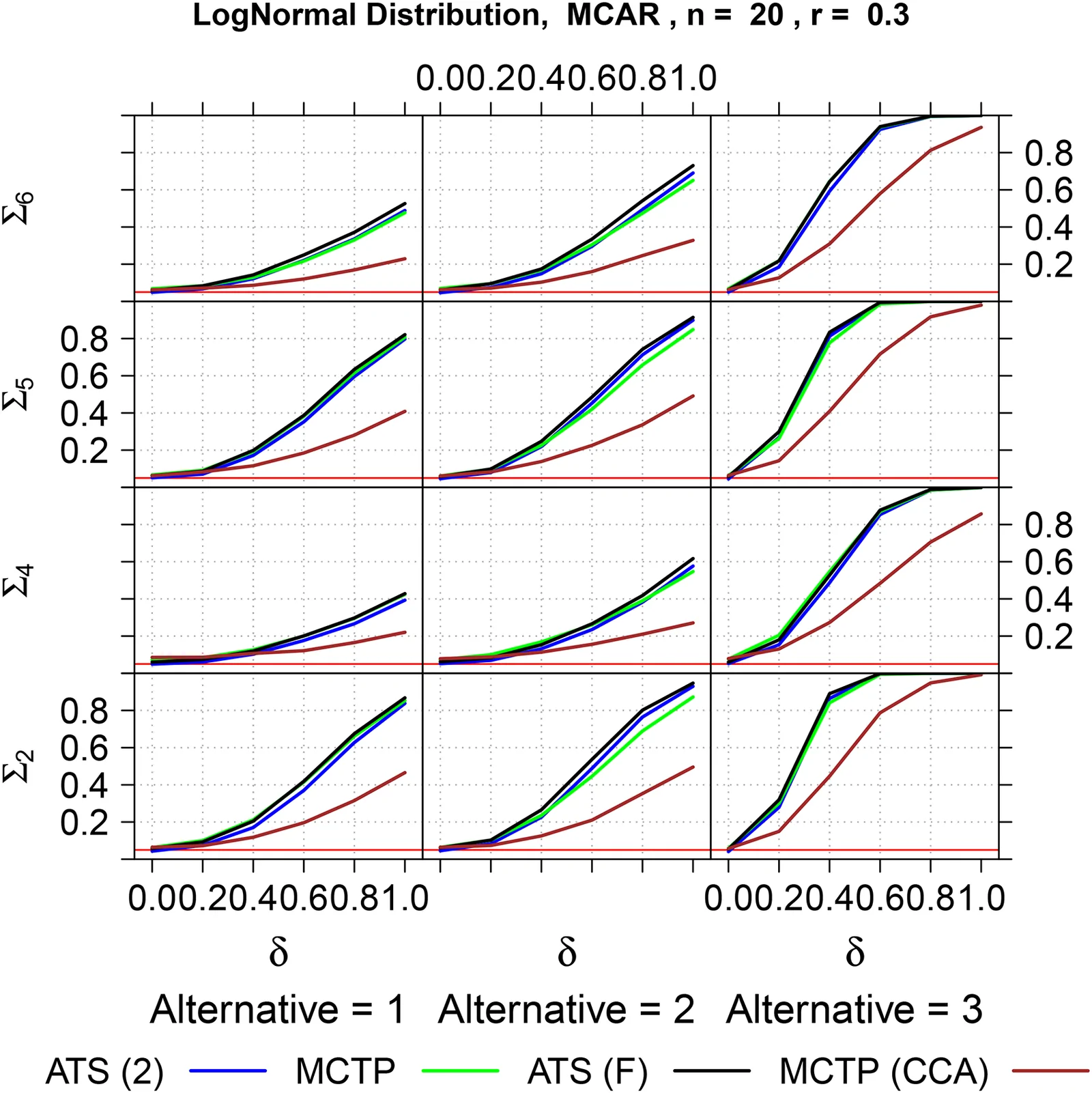
Power simulation of the second version of the ATS and the MCTP for testing $H_{0}^{p}$ , the ATS for testing $H_{0}^{F}$ and the MCTP for testing $H_{0}^{p}$ using only complete cases, data is MCAR. MCTP: multiple contrast test procedure; MCAR: missing completely at random; ATS: ANOVA-type statistic.

Next, we investigate the power of the procedures under both MAR scenarios. The results can be found in Figures S. 2 to S. 5. Similar to the results under MCAR, none of the procedures which use all-available information is superior. However, the MCTP using only complete cases exhibits a comparable power in the second MAR scenario compared to the competing procedures using all-available information. To summarize, we still recommend the novel MCTP for testing $H_{0}^{p}$ using all-availabe information if data is MAR.

## 9 Analysis of the example

The headache severity level migraine trial presented in Section “Motivating example” was analyzed using the MCTP, since this procedure can be used not only for testing the global hypothesis of no effect over all time points but also for testing pairwise comparisons. Thus, we chose a Tukey-type contrast matrix

$$C=\left(\begin{array}{c} c_{1}^{\prime }\\ c_{2}^{\prime }\\ c_{3}^{\prime }\\ c_{4}^{\prime }\\ c_{5}^{\prime }\\ c_{6}^{\prime } \end{array}\right)=\left(\begin{array}{cccc} -1 & 1 & 0 & 0\\ -1 & 0 & 1 & 0\\ -1 & 0 & 0 & 1\\ 0 & -1 & 1 & 0\\ 0 & -1 & 0 & 1\\ 0 & 0 & -1 & 1 \end{array}\right)$$

for testing the null hypotheses $H_{0}^{p}\;:\;Cp=0$ . The estimated unweighted relative effects are given by $\hat{p}=({\hat{\;p}}_{1},{\hat{\;p}}_{2},{\hat{\;p}}_{3},{\hat{\;p}}_{4}{)}^{\prime }=(0.55,0.47,0.45,0.53{)}^{\prime }$ , which indicate that the scores obtained under session 3 are smallest, followed by sessions 2 and 4, whereas the scores obtained under session 1 are the largest. These computations match the visual impression attained by the boxplots in Figure 1. As the direction of the trend was unknown, we calculated two-sided simultaneous confidence intervals (22) at 95% confidence level. The results along with the values of test statistics $T_{\ell }$ and *p*-values are displayed in Table 2. Note, that no multiplicity adjustment was necessary as we used the critical value obtained from the MCTP in each comparison.

It follows from Table 2 that the data provides the evidence to reject the global null hypothesis $H_{0}^{p}$ (p-val. = 0.022 ), indicating that the treatment has an effect on the migraine of the patients over the course of time. However, session 4 does not indicate an improvement upon the first session. Applying the ANOVA-type procedure yields a higher global p-value of 0.047 (first version of the ATS) and 0.049 (second version of the ATS), respectively.

**Table 2.** Point estimators, simultaneous confidence intervals, *t*-values, and *p*-values for Tukey-type contrasts in relative effects in the migraine trial.

Comparison	Estimator	95 %- Confidence interval	*t*-value	*p*-value
${\hat{\;p}}_{2}-{\hat{\;p}}_{1}$	−0.087	[ −0.165 , −0.016 ]	2.867	0.023
${\hat{\;p}}_{3}-{\hat{\;p}}_{1}$	−0.103	[ −0.196 , −0.020 ]	2.882	0.022
${\hat{\;p}}_{4}-{\hat{\;p}}_{1}$	−0.028	[ −0.148 , 0.080]	0.610	0.927
${\hat{\;p}}_{3}-{\hat{\;p}}_{2}$	−0.017	[ −0.099 , 0.058]	0.522	0.952
${\hat{\;p}}_{4}-{\hat{\;p}}_{2}$	0.058	[ −0.063 0.168]	1.241	0.594
${\hat{\;p}}_{4}-{\hat{\;p}}_{3}$	0.075	[ −0.049 , 0.187]	1.566	0.393

## 10 Discussion and conclusions

Missing values appear naturally in RM designs. Beyond proper statistical modeling, estimation problems of (model) parameters exist and typically complicate the analysis. In this paper, we discussed purely nonparametric methods and their limitations. In particular, we extended the methods proposed by Konietschke et al.^5^ to allow for missing data. The simulation study demonstrates that the proposed methodology controls the type-I-error satisfactorily even for small sample sizes and a high missing rate. The already existing method for RM data with missing values from Domhof et al.^3^ can only be used for testing the null hypothesis $H_{0}^{F}\;:\;F_{1}=\cdots =F_{d}$ with regard to the marginal distribution functions, which is difficult to interpret and does not allow for calculating confidence intervals. The newly proposed method can be used for testing the less strict hypothesis $H_{0}^{p}$ and for calculating confidence intervals. Note, that the procedures are not limited to metric data; even ordered categorical data and binary data can be examined in a unified way. All results achieved in this paper are valid under the MCAR mechanism and the results of our simulation study indicate that all proposed methods are not sensitive to MAR scenarios. Further simulation studies indicated that in “extreme” scenarios, for example, smaller sample sizes, high missing probabilities, heteroscedasticity, and high correlations, the MCTP tends to be liberal. Therefore, we plan to explore resampling techniques for making the MCTP more accurate in these scenarios. Moreover, extensions to split plot designs and clustered data will be part of future research.

## Supplemental Material

sj-pdf-2-smm-10.1177_09622802211046389 - Supplemental material for Ranking procedures for repeated measures designs with missing data: Estimation, testing and asymptotic theory Supplemental material, sj-pdf-2-smm-10.1177_09622802211046389 for Ranking procedures for repeated measures designs with missing data: Estimation, testing and asymptotic theory by Kerstin Rubarth, Markus Pauly and Frank Konietschke in Statistical Methods in Medical Research
