# Supplementary material for: Ranking procedures for repeated measures designs with missing data: Estimation, testing and asymptotic theory
Source: Stat Methods Med Res. 2021 Nov 29;31(1):105–18. doi: 10.1177/09622802211046389 (PMC8721540; doi:10.1177/09622802211046389)
Supplement: sj-pdf-2-smm-10.1177_09622802211046389 - Supplemental material for Ranking procedures for repeated measures designs with missing data: Estimation, testing and asymptotic theory [file sj-pdf-2-smm-10.1177_09622802211046389.pdf]

Journal Title
XX(X):1–20
©The Author(s) 2016
Reprints and permission:
sagepub.co.uk/journalsPermissions.nav
DOI: 10.1177/ToBeAssigned
www.sagepub.com/
SAGE

Supplementary Material to: Ranking Procedures for Repeated Measures Designs with Missing Data - Estimation, Testing and Asymptotic Theory

Kerstin Rubarth [1], Markus Pauly [2] and Frank Konietschke[1]

We present in this supplementary material selected results from the type-I error and power simulations. Furthermore, we present the proofs of all theorems and propositions from the paper.

1 Results of the Simulation Study

Within this section, we will present additional tables and figures of the type-I error and power results of the simulation study. We simulated

1. the WTS in (16) with a critical value from a $\chi^2_{\hat{f}}$ distribution,

[1]Charité - Universitätsmedizin Berlin, Corporate Member of Freie Universität Berlin, Humboldt-Universität zu Berlin, and Berlin Institute of Health, Institute of Biometry and Clinical Epidemiolgy, Chariteplatz 1, 10117 Berlin, Germany
[2]TU Dortmund University, Department of Statistics, 44221 Dortmund, Germany

Corresponding author:
Frank Konietschke, Charité - Universitätsmedizin Berlin, Corporate Member of Freie Universität Berlin, Humboldt-Universität zu Berlin, and Berlin Institute of Health, Institute of Biometry and Clinical Epidemiolgy, Chariteplatz 1, 10117 Berlin, Germany
Email: frank.konietschke@charite.de

2. the ATS (1) in (17) with the proposed F-approximation,
3. the ATS (2) in (18) with the proposed F-approximation,
4. the MCTP T_0 in (19) with a $t_{n-1}(\mathbf{0}, \widehat{\boldsymbol{R}}_n)$ approximation,

and compared them with

5. the WTS and ATS statistics for testing H_0^F as proposed by Domhof et al.[1]

in different homo- and heteroscedastic repeated measures designs with different rates of missing values. Even though Domhof et al.[1] reported a liberal behavior of the WTS (for testing H_0^F), we added the method as a competing procedure for completeness. We thus also investigated their robustness to variance heteroscedasticity. Since all of the methods above use all-available data, we additionally compared them with two MCTP-based approaches: a complete case analysis and a naive imputation approach, in which we either

6. deleted the whole observation vector $\boldsymbol{X}_k$ of subject k if any X_{ik} was missing ($\lambda_{ik} = 0$), or
7. if X_{ik} was missing ($\lambda_{ik} = 0$), we calculated $median(\lambda_{i1}X_{i1}, \ldots, \lambda_{in}X_{in})$, and assigned it to X_{ik} and set $\lambda_{ik} = 1$.

Data have been generated using discretized, by rounding to integers, normal and log-normal distributions with varying numbers of time points $d \in \{3, 4\}$, sample sizes $n \in \{20, 30, 50\}$, amount of missing values $r \in \{0\%, 10\%, 30\%\}$ and six different types of covariance matrices

$$\boldsymbol{\Sigma}_1 = \begin{pmatrix} 1 & 0.5 & 0.5 \\ 0.5 & 1 & 0.5 \\ 0.5 & 0.5 & 1 \end{pmatrix}, \qquad \boldsymbol{\Sigma}_2 = \begin{pmatrix} 1 & 0.5 & 0.5 & 0.5 \\ 0.5 & 1 & 0.5 & 0.5 \\ 0.5 & 0.5 & 1 & 0.5 \\ 0.5 & 0.5 & 0.5 & 1 \end{pmatrix}, \tag{1}$$

$$\boldsymbol{\Sigma}_3 = \begin{pmatrix} 1 & 0.3 & 0.6 \\ 0.3 & 1.2 & 0.9 \\ 0.6 & 0.9 & 1.5 \end{pmatrix}, \qquad \boldsymbol{\Sigma}_4 = \begin{pmatrix} 1 & 0.2 & 0.4 & 0.6 \\ 0.2 & 2 & 0.7 & 0.5 \\ 0.4 & 0.7 & 2.5 & 0.6 \\ 0.6 & 0.5 & 0.6 & 3 \end{pmatrix},$$

$$\boldsymbol{\Sigma}_5 = \begin{pmatrix} 1 & 0.6 & 0.36 & 0.216 \\ 0.6 & 1 & 0.6 & 0.36 \\ 0.36 & 0.6 & 1 & 0.6 \\ 0.216 & 0.36 & 0.6 & 1 \end{pmatrix}, \qquad \boldsymbol{\Sigma}_6 = \begin{pmatrix} 1 & 0.8 & 0.64 & 0.512 \\ 0.8 & 1.5 & 0.8 & 0.64 \\ 0.64 & 0.8 & 2 & 0.8 \\ 0.512 & 0.64 & 0.8 & 2.5 \end{pmatrix}.$$

The covariance matrices were chosen to model a broad selection of dependency patterns, including homoscedastic ($\boldsymbol{\Sigma}_1$ and $\boldsymbol{\Sigma}_2$) as well as heteroscedastic marginals. Note that H_0^F holds only under $\boldsymbol{\Sigma}_1$ and $\boldsymbol{\Sigma}_2$. We furthermore investigate the methods sensitivity to both *MCAR* and *MAR* data to cover realistic scenarios. In order to generate the former, we multiplied the observations with randomly chosen indicators $\lambda_{ik} \sim B(1-r)$, with

a zero entry being interpreted as a missing observation, whereas we followed Santos et. al[2] for the latter. Hereby we defined pairs of observations $\{X_{obs}, X_{miss}\}$, where X_{obs} determines the probability that X_{miss} was actually observed. For instance, in case of $d = 4$ we defined the pairs $\{X_{1k}, X_{2k}\}$ and $\{X_{3k}, X_{4k}\}$. Following the idea of Amro et al.[3], we investigated two different types of MAR scenarios, MAR (1) and MAR (2). First, for the MAR (1) scenario, we divided $X_{i,obs}$ into three groups: (1) $\{\boldsymbol{X}_{ik} = X_{i,obs} \in (-\infty, -\sigma_i), k = 1, \dots, n\}$, (2) $\{\boldsymbol{X}_{ik} = X_{i,obs} \in (-\sigma_i, \sigma_i), k = 1, \dots, n\}$ and (3) $\{\boldsymbol{X}_{ik} = X_{i,obs} \in (\sigma_i, \infty), k = 1, \dots, n\}$, where σ_i^2 is the variance of $X_{i,obs}$. Then, we assigned a missing rate of 10% to the first and third group and a missing rate of 30% to the second group. Second, in the MAR (2) scenario, data was divided into two groups using the median, following the idea of Zhu et al[4]. Specifically, we defined (1) $\{\boldsymbol{X}_{ik} = X_{i,obs} \in (-\infty, median(X_{i,obs}), k = 1, ..., n\}$ and (2) $\{\boldsymbol{X}_{ik} = X_{i,obs} \in (median(X_{i,obs}), \infty), k = 1, ..., n\}$. Here, we assigned a missing rate of 0% to the first group and a missing rate of 10% to the second group.

In order to investigate the power of the procedures, a simulation study was conducted using four-dimensional normal and log-normal distributions with $\boldsymbol{\mu} = (\mu_1, \mu_2, \mu_3, \mu_4)'$ and covariance matrices $\boldsymbol{\Sigma}_2, \boldsymbol{\Sigma}_4, \boldsymbol{\Sigma}_5, \boldsymbol{\Sigma}_6$. In particular, three different types of shift-alternatives were considered

Alternative 1	Alternative 2	Alternative 3	
$\boldsymbol{\mu} = (0, 0, 0, \delta)'$	$\boldsymbol{\mu} = (0, 0, \delta, \delta)'$	$\boldsymbol{\mu} = (0, 1\delta, 2\delta, 3\delta)'$,	(2)

with ranging $\delta = (0.2, 0.4, 0.6, 0.8, 1, 1.5)$ and different amount of missing values. As the WTS turned out to be inappropriate for small sample sizes, it was not included into the power analysis. Moreover, since the second version of the ATS for testing H_0^p showed a more accurate behaviour than the first version, we only present results for the second version.

For each design, $10,000$ simulation runs were performed using the *R* software package of statistical computing, version R 3.6.4[5]. The complete simulation code is available on https://github.com/KerstinRubarth/RM_Miss.

1.1 Type-I error

The type-I error rates under the MCAR mechanism for different sample sizes n, covariances matrices, missing rates r and discretized distributions can be found in Tables 1 - 3. A graphical comparisons of rather small and large sample sizes and no, medium and high missing rates can be found in Figure 1.

Table 1. Type-I-error ($\alpha = 5\%$) simulation results of the ATS and WTS for testing H_0^F and H_0^p and MCTP in different designs with covariance matrices $\mathbf{\Sigma}_1$ and $\mathbf{\Sigma}_2$ as given in (23). Here, r denotes the rate of missing data. Data is MCAR. Note that H_0^F holds.

			$\mathbf{\Sigma}_1$						$\mathbf{\Sigma}_2$					
			H_0^F		H_0^p				H_0^F		H_0^p			
n	r(%)	Dist	WTS	ATS	WTS	ATS1	ATS2	MCT	WTS	ATS	WTS	ATS1	ATS2	MCT
20	0	N	0.102	0.066	0.075	0.051	0.038	0.047	0.141	0.062	0.098	0.047	0.038	0.051
20	10	N	0.097	0.066	0.078	0.053	0.043	0.049	0.138	0.060	0.104	0.050	0.041	0.054
20	30	N	0.099	0.061	0.090	0.058	0.046	0.058	0.137	0.058	0.118	0.056	0.045	0.061
30	0	N	0.086	0.062	0.070	0.054	0.046	0.052	0.102	0.056	0.082	0.047	0.040	0.049
30	10	N	0.081	0.059	0.070	0.051	0.043	0.051	0.100	0.056	0.083	0.049	0.042	0.052
30	30	N	0.080	0.059	0.077	0.057	0.048	0.058	0.104	0.057	0.095	0.054	0.047	0.059
50	0	N	0.068	0.056	0.061	0.052	0.047	0.049	0.075	0.051	0.066	0.047	0.043	0.051
50	10	N	0.066	0.056	0.061	0.052	0.047	0.049	0.075	0.052	0.067	0.048	0.044	0.048
50	30	N	0.068	0.055	0.065	0.053	0.048	0.054	0.079	0.056	0.075	0.054	0.050	0.056
20	0	LN	0.100	0.059	0.074	0.046	0.037	0.048	0.139	0.060	0.097	0.045	0.036	0.050
20	10	LN	0.096	0.059	0.076	0.049	0.038	0.047	0.142	0.060	0.109	0.050	0.040	0.053
20	30	LN	0.096	0.060	0.087	0.057	0.046	0.059	0.140	0.058	0.117	0.054	0.043	0.062
30	0	LN	0.082	0.060	0.068	0.051	0.043	0.050	0.100	0.054	0.081	0.047	0.041	0.049
30	10	LN	0.085	0.062	0.073	0.056	0.046	0.053	0.097	0.055	0.082	0.047	0.041	0.052
30	30	LN	0.077	0.057	0.074	0.056	0.048	0.054	0.095	0.050	0.088	0.047	0.040	0.054
50	0	LN	0.063	0.052	0.058	0.048	0.044	0.047	0.075	0.052	0.066	0.047	0.043	0.048
50	10	LN	0.066	0.053	0.061	0.049	0.045	0.050	0.079	0.054	0.072	0.049	0.045	0.050
50	30	LN	0.069	0.056	0.067	0.054	0.049	0.055	0.079	0.053	0.076	0.052	0.048	0.054

Table 2. Type-I-error ($\alpha = 5\%$) simulation results of the ATS and WTS for testing H_0^F and H_0^p and MCTP in different designs with covariance matrices $\mathbf{\Sigma}_3$ and $\mathbf{\Sigma}_4$ as given in (23). Here, r denotes the rate of missing data. Data is MCAR. Note that H_0^p holds.

			$\mathbf{\Sigma}_3$						$\mathbf{\Sigma}_4$					
			H_0^F		H_0^p				H_0^F		H_0^p			
n	r(%)	Dist	WTS	ATS	WTS	ATS1	ATS2	MCT	WTS	ATS	WTS	ATS1	ATS2	MCT
20	0	N	0.111	0.066	0.078	0.053	0.043	0.050	0.130	0.058	0.108	0.047	0.038	0.054
20	10	N	0.102	0.065	0.077	0.054	0.044	0.049	0.134	0.060	0.118	0.055	0.045	0.060
20	30	N	0.100	0.064	0.088	0.059	0.048	0.059	0.140	0.062	0.133	0.062	0.051	0.070
30	0	N	0.087	0.063	0.065	0.052	0.044	0.050	0.100	0.053	0.087	0.048	0.041	0.051
30	10	N	0.082	0.061	0.069	0.053	0.045	0.051	0.099	0.054	0.090	0.050	0.043	0.053
30	30	N	0.084	0.061	0.078	0.057	0.050	0.058	0.105	0.059	0.099	0.057	0.051	0.066
50	0	N	0.071	0.057	0.061	0.052	0.048	0.049	0.082	0.056	0.071	0.051	0.047	0.051
50	10	N	0.070	0.056	0.062	0.051	0.047	0.050	0.080	0.055	0.072	0.051	0.048	0.057
50	30	N	0.069	0.055	0.066	0.053	0.048	0.054	0.083	0.057	0.080	0.055	0.051	0.060
20	0	LN	0.105	0.064	0.074	0.051	0.041	0.046	0.136	0.058	0.113	0.052	0.042	0.056
20	10	LN	0.107	0.070	0.083	0.058	0.047	0.055	0.130	0.056	0.115	0.051	0.040	0.061
20	30	LN	0.100	0.063	0.088	0.060	0.047	0.058	0.141	0.060	0.130	0.060	0.049	0.073
30	0	LN	0.089	0.061	0.068	0.053	0.044	0.049	0.105	0.059	0.093	0.053	0.046	0.056
30	10	LN	0.084	0.063	0.073	0.056	0.047	0.052	0.110	0.064	0.102	0.060	0.052	0.065
30	30	LN	0.080	0.058	0.075	0.055	0.047	0.056	0.108	0.058	0.109	0.058	0.049	0.067
50	0	LN	0.070	0.054	0.060	0.048	0.044	0.049	0.087	0.058	0.080	0.053	0.050	0.058
50	10	LN	0.066	0.056	0.059	0.052	0.047	0.049	0.087	0.059	0.082	0.055	0.050	0.059
50	30	LN	0.069	0.060	0.066	0.058	0.054	0.054	0.087	0.062	0.085	0.060	0.055	0.063

Table 3. Type-I-error ($\alpha = 5\%$) simulation results of the ATS and WTS for testing H_0^F and H_0^p and MCTP in different designs with covariance matrices $\mathbf{\Sigma}_5$ and $\mathbf{\Sigma}_6$ as given in (23). Here, r denotes the rate of missing data. Data is MCAR. Note that H_0^p holds.

			$\mathbf{\Sigma}_5$						$\mathbf{\Sigma}_6$					
			H_0^F		H_0^p				H_0^F		H_0^p			
n	r(%)	Dist	WTS	ATS	WTS	ATS1	ATS2	MCT	WTS	ATS	WTS	ATS1	ATS2	MCT
20	0	N	0.141	0.058	0.097	0.051	0.042	0.049	0.147	0.060	0.101	0.050	0.041	0.052
20	10	N	0.139	0.057	0.104	0.051	0.042	0.052	0.146	0.062	0.111	0.053	0.042	0.058
20	30	N	0.138	0.057	0.119	0.055	0.045	0.064	0.147	0.063	0.125	0.060	0.051	0.068
30	0	N	0.100	0.054	0.080	0.048	0.042	0.046	0.114	0.059	0.087	0.052	0.044	0.049
30	10	N	0.097	0.052	0.081	0.048	0.040	0.048	0.102	0.055	0.083	0.049	0.041	0.050
30	30	N	0.102	0.056	0.094	0.055	0.047	0.058	0.104	0.060	0.094	0.057	0.049	0.058
50	0	N	0.076	0.054	0.066	0.052	0.048	0.050	0.083	0.055	0.069	0.050	0.045	0.051
50	10	N	0.074	0.052	0.067	0.049	0.044	0.051	0.080	0.053	0.070	0.049	0.045	0.050
50	30	N	0.074	0.050	0.073	0.049	0.045	0.056	0.078	0.052	0.073	0.049	0.045	0.053
20	0	LN	0.141	0.060	0.101	0.049	0.040	0.050	0.148	0.064	0.107	0.054	0.043	0.054
20	10	LN	0.138	0.059	0.109	0.051	0.041	0.054	0.139	0.062	0.104	0.054	0.046	0.054
20	30	LN	0.142	0.062	0.124	0.061	0.051	0.066	0.142	0.059	0.122	0.058	0.049	0.068
30	0	LN	0.102	0.060	0.084	0.054	0.048	0.052	0.110	0.064	0.089	0.055	0.047	0.054
30	10	LN	0.096	0.051	0.079	0.046	0.040	0.051	0.104	0.063	0.088	0.058	0.050	0.056
30	30	LN	0.098	0.054	0.091	0.053	0.047	0.059	0.100	0.056	0.093	0.054	0.047	0.056
50	0	LN	0.080	0.058	0.070	0.054	0.050	0.054	0.088	0.063	0.077	0.057	0.052	0.057
50	10	LN	0.075	0.052	0.069	0.050	0.046	0.049	0.084	0.060	0.073	0.055	0.051	0.056
50	30	LN	0.075	0.051	0.072	0.051	0.046	0.054	0.079	0.057	0.075	0.055	0.051	0.054

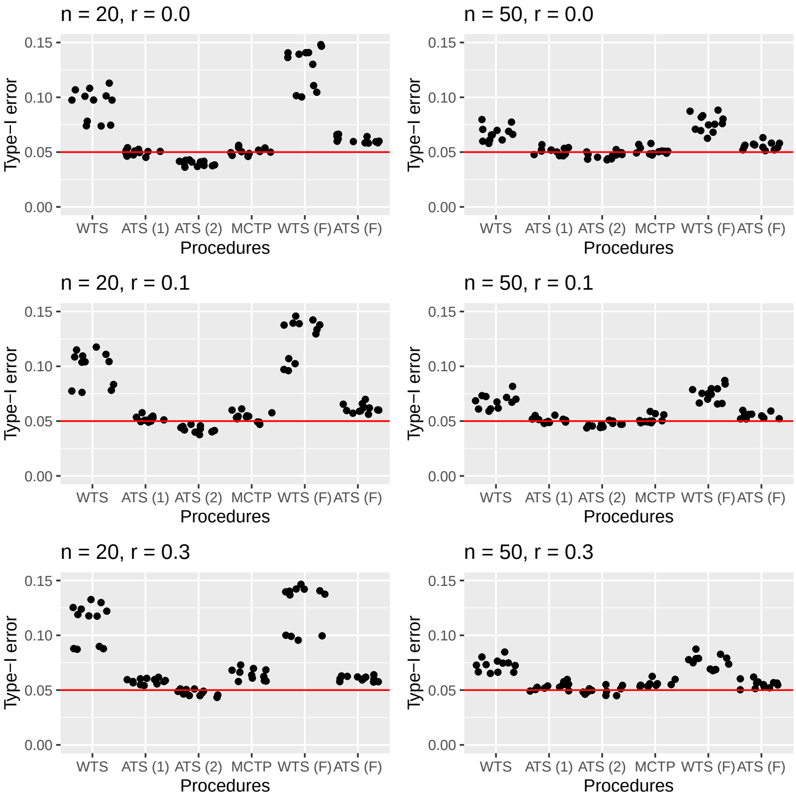

Figure 1. Type-I error rates of the newly proposed Wald- (WTS), ANOVA-type (ATS (1) and ATS (2)) and MCT procedures and the procedures of Domhof et al.[1] in MCAR scenarios.

1.2 Power

The power simulation results for $n = 30$ and Σ_4 are given in Tables 4 and 5 for discretized normal and log-normal distributions respectively. An exploration of the power under different MAR scenarios, covariance matrices, alternatives and discretized distributions can be found in Figures 2 - 5.

Table 4. Power comparisons of the ATS for testing either H_0^F (ATS (F)) or H_0^p (ATS (2))and the MCTP to detect the three alternatives listed in (24) in normal distributions with $\mathbf{\Sigma}_4$ and $n = 30$. Data is MCAR.

		Alternative 1			Alternative 2			Alternative 3		
delta	r(%)	ATS(F)	ATS(2)	MCTP	ATS(F)	ATS(2)	MCTP	ATS(F)	ATS(2)	MCTP
0.0	0	0.053	0.041	0.051	0.059	0.047	0.056	0.055	0.042	0.050
0.0	10	0.054	0.043	0.053	0.058	0.046	0.058	0.057	0.043	0.058
0.0	30	0.059	0.051	0.066	0.056	0.047	0.061	0.057	0.049	0.061
0.2	0	0.085	0.070	0.078	0.099	0.079	0.095	0.309	0.267	0.301
0.2	10	0.080	0.067	0.077	0.088	0.072	0.087	0.269	0.234	0.269
0.2	30	0.074	0.064	0.081	0.082	0.071	0.093	0.208	0.189	0.227
0.4	0	0.176	0.150	0.152	0.248	0.212	0.220	0.903	0.880	0.872
0.4	10	0.160	0.138	0.149	0.219	0.186	0.201	0.851	0.821	0.823
0.4	30	0.132	0.118	0.129	0.177	0.157	0.175	0.694	0.666	0.688
0.6	0	0.355	0.313	0.317	0.526	0.481	0.430	0.999	0.999	0.998
0.6	10	0.319	0.284	0.286	0.454	0.411	0.377	0.997	0.995	0.993
0.6	30	0.235	0.212	0.227	0.341	0.314	0.311	0.974	0.968	0.966
0.8	0	0.574	0.532	0.527	0.801	0.766	0.682	1.000	1.000	1.000
0.8	10	0.521	0.484	0.474	0.732	0.694	0.617	1.000	1.000	1.000
0.8	30	0.391	0.367	0.376	0.572	0.540	0.493	1.000	0.999	0.999
1.0	0	0.789	0.755	0.742	0.949	0.936	0.862	1.000	1.000	1.000
1.0	10	0.720	0.685	0.676	0.912	0.896	0.821	1.000	1.000	1.000
1.0	30	0.566	0.540	0.546	0.784	0.758	0.693	1.000	1.000	1.000
1.5	0	0.987	0.983	0.982	1.000	1.000	0.998	1.000	1.000	1.000
1.5	10	0.974	0.967	0.964	1.000	0.999	0.994	1.000	1.000	1.000
1.5	30	0.904	0.891	0.886	0.988	0.986	0.965	1.000	1.000	1.000

Table 5. Power comparisons of the ATS for testing either H_0^F (ATS (F)) or H_0^p (ATS (2))and the MCTP to detect the three alternatives listed in (24) in log-normal distributions with $\mathbf{\Sigma}_4$ and $n = 30$. Data is MCAR.

		Alternative 1			Alternative 2			Alternative 3		
delta	r(%)	ATS(F)	ATS(2)	MCTP	ATS(F)	ATS(2)	MCTP	ATS(F)	ATS(2)	MCTP
0.0	0	0.059	0.046	0.056	0.059	0.047	0.060	0.059	0.048	0.057
0.0	10	0.064	0.052	0.065	0.064	0.052	0.066	0.059	0.047	0.061
0.0	30	0.058	0.049	0.067	0.059	0.050	0.065	0.061	0.053	0.066
0.2	0	0.100	0.083	0.094	0.124	0.102	0.121	0.366	0.329	0.366
0.2	10	0.099	0.082	0.093	0.110	0.091	0.108	0.337	0.300	0.335
0.2	30	0.085	0.074	0.088	0.098	0.086	0.107	0.238	0.214	0.260
0.4	0	0.205	0.175	0.184	0.303	0.263	0.261	0.926	0.906	0.904
0.4	10	0.188	0.162	0.166	0.260	0.229	0.237	0.880	0.857	0.859
0.4	30	0.143	0.127	0.145	0.207	0.186	0.199	0.731	0.704	0.732
0.6	0	0.397	0.357	0.350	0.581	0.536	0.479	0.999	0.999	0.999
0.6	10	0.345	0.312	0.316	0.505	0.465	0.428	0.998	0.997	0.996
0.6	30	0.269	0.246	0.262	0.387	0.358	0.354	0.977	0.973	0.973
0.8	0	0.619	0.579	0.569	0.830	0.798	0.715	1.000	1.000	1.000
0.8	10	0.548	0.511	0.504	0.764	0.732	0.658	1.000	1.000	1.000
0.8	30	0.423	0.397	0.408	0.608	0.579	0.535	0.999	0.999	0.999
1.0	0	0.809	0.777	0.769	0.959	0.945	0.887	1.000	1.000	1.000
1.0	10	0.746	0.714	0.703	0.932	0.917	0.854	1.000	1.000	1.000
1.0	30	0.596	0.573	0.574	0.813	0.792	0.729	1.000	1.000	1.000
1.5	0	0.991	0.988	0.986	1.000	1.000	0.999	1.000	1.000	1.000
1.5	10	0.977	0.971	0.965	1.000	0.999	0.996	1.000	1.000	1.000
1.5	30	0.916	0.906	0.898	0.993	0.992	0.975	1.000	1.000	1.000

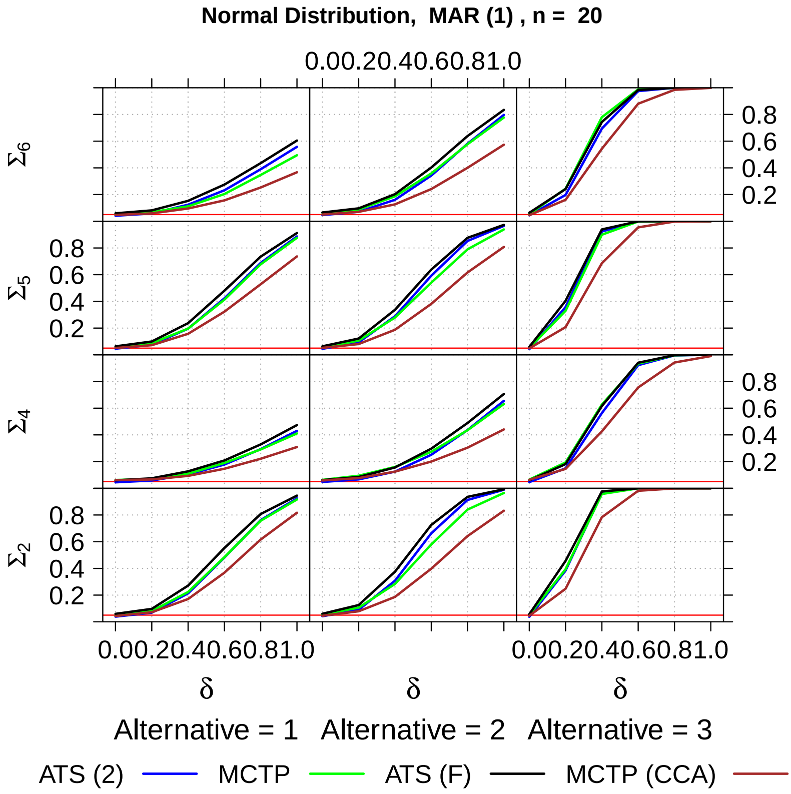

Figure 2. Power simulation of the ATS (2) and the MCTP for testing H_0^p, the ATS for testing H_0^F and the MCTP for testing H_0^p using only complete cases, data is MAR (1).

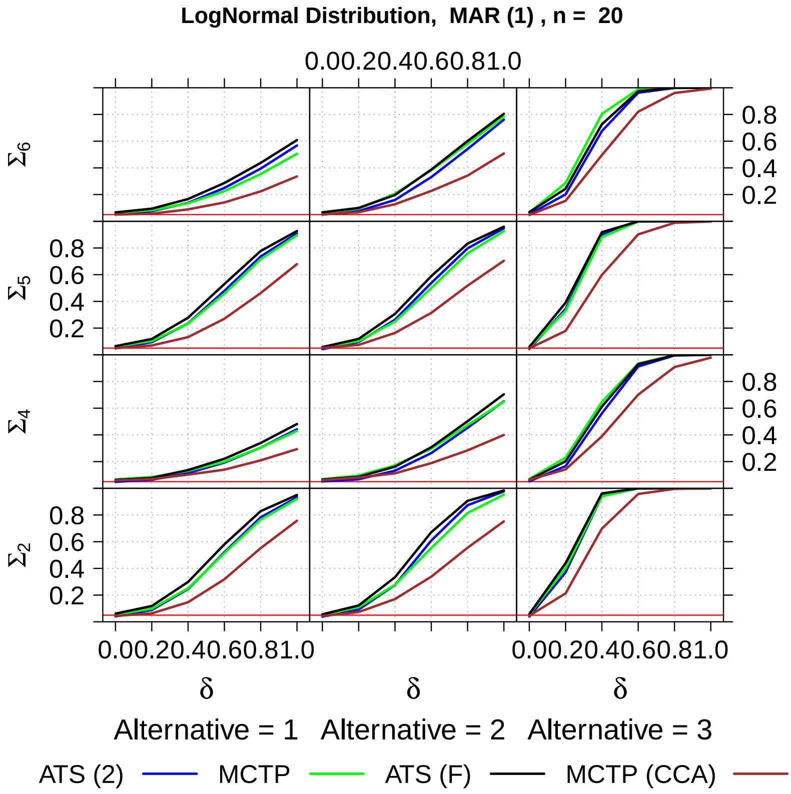

Figure 3. Power simulation of the ATS (2) and the MCTP for testing H_0^p, the ATS for testing H_0^F and the MCTP for testing H_0^p using only complete cases, data is MAR (1).

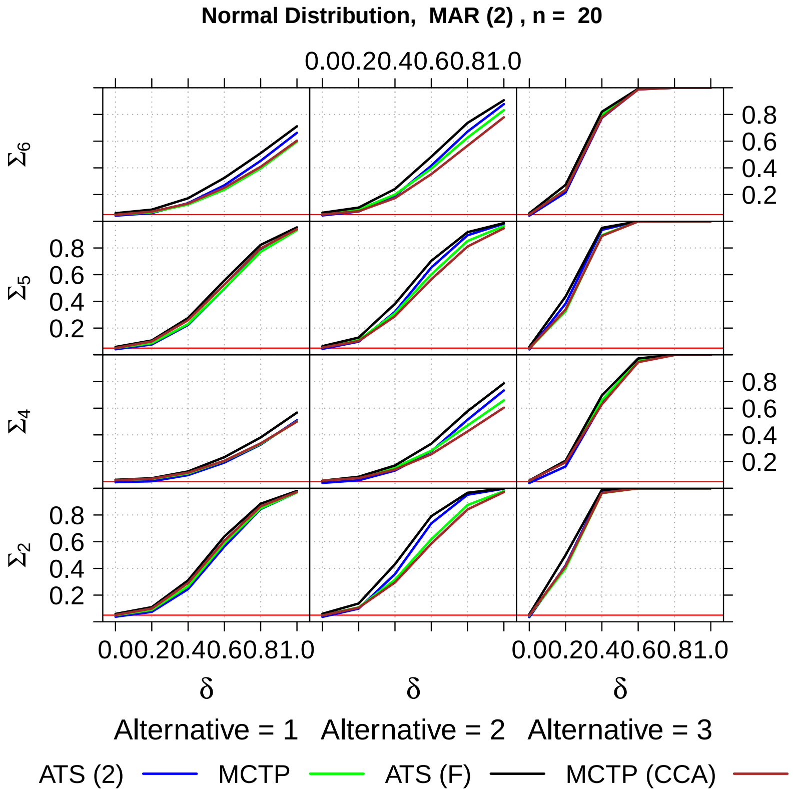

Figure 4. Power simulation of the ATS (2) and the MCTP for testing H_0^p, the ATS for testing H_0^F and the MCTP for testing H_0^p using only complete cases, data is MAR (2).

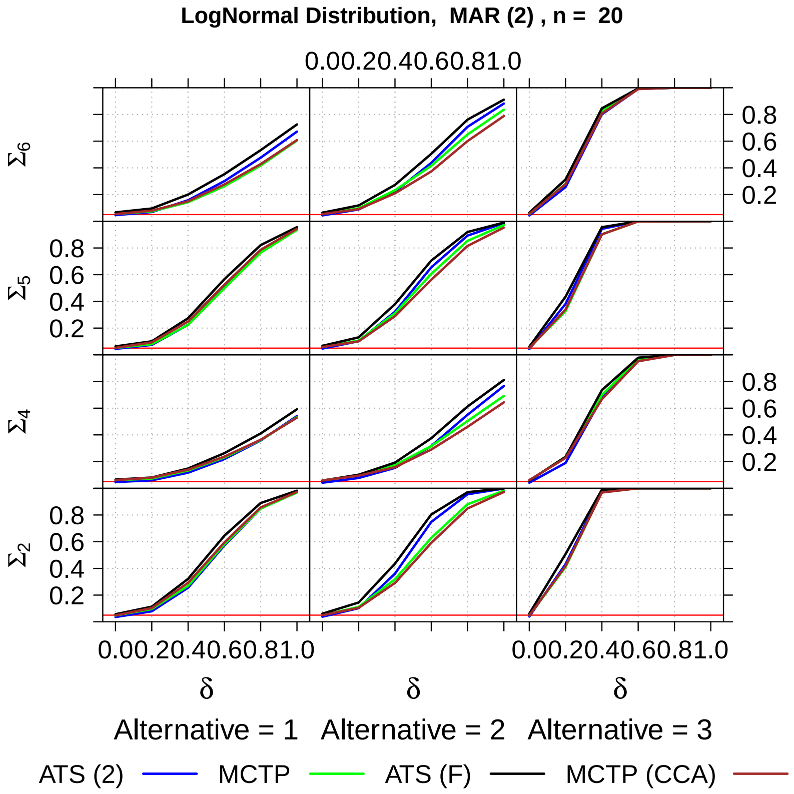

Figure 5. Power simulation of the ATS (2) and the MCTP for testing H_0^p, the ATS for testing H_0^F and the MCTP for testing H_0^p using only complete cases, data is MAR (2).

2 Proofs

In this section we will provide the proofs of the theoretical results achieved in the paper. The strong consistency of the point and variance estimators follows from the following generalization of the Glivenko-Cantelli Theorem:

Lemma 1: Let $F_i^{(-)}(x) = P(X_{i1} < x)$ and $F_i^{(+)}(x) = P(X_{i1} \leq x)$ denote the left-continuous and right-continuous versions of the distribution function of X_{i1} and let

$$\widehat{F}_i^{(-)} = \frac{1}{\lambda_i} \sum_{k=1}^{n} \lambda_{ik} c^{(-)}(x - X_{ik}) \text{ and } \widehat{F}_i^{(+)} = \frac{1}{\lambda_i} \sum_{k=1}^{n} \lambda_{ik} c^{(+)}(x - X_{ik}) \tag{3}$$

denote their empirical counterparts, where

$$c^{(-)}(u) = \begin{cases} 0 & u \leq 0 \\ 1 & u > 0 \end{cases} \quad \text{and} \quad c^{(+)}(u) = \begin{cases} 0 & u < 0 \\ 1 & u \geq 0 \end{cases} .$$

Furthermore, let $F_i(x) = \frac{1}{2}(F_i^{(-)}(x) + F_i^{(+)}(x))$, $G(x)$, $\widehat{F}_i(x) = \frac{1}{2}(\widehat{F}_i^{(-)} + \widehat{F}_i^{(+)})$ and $\widehat{G}(x) = \frac{1}{d}\sum_{i=1}^{d} \widehat{F}_i(x)$ denote the normalized versions of the distribution functions as used in the manuscript. Then,

$$\left|\left|\widehat{F}_i - F_i\right|\right|_\infty \overset{a.s.}{\to} 0,\ \lambda_i \to \infty \ \text{ and } \ \left|\left|\widehat{G} - G\right|\right|_\infty \overset{a.s.}{\to} 0,\ \min\{\lambda_1, \ldots, \lambda_d\} \to \infty.$$

Proof: Since $\widehat{F}_i(x)$ is the mean of the left- and right continuous version, it follows

$$\begin{aligned}\left|\left|\widehat{F}_i - F_i\right|\right|_\infty &= \left|\left|\frac{1}{2}(\widehat{F}_i^{(-)} - F_i^{(-)}) + \frac{1}{2}(\widehat{F}_i^{(+)} - F_i^{(+)})\right|\right|_\infty \\ &\leq \frac{1}{2}\left|\left|\widehat{F}_i^{(-)} - F_i^{(-)}\right|\right|_\infty + \frac{1}{2}\left|\left|\widehat{F}_i^{(+)} - F_i^{(+)}\right|\right|_\infty \overset{a.s.}{\to} 0, \lambda_i \to \infty.\end{aligned}$$

The proof of the almost sure convergence of the last remaining terms is given by[6], page 111. Furthermore, by triangle inequality, the convergence $\left|\left|\widehat{G} - G\right|\right|_\infty \overset{a.s.}{\to} 0$ follows, which completes the proof.

2.1 Proof of Proposition 1

1. The estimator $\widehat{\boldsymbol{p}} = (\widehat{p}_1, ..., \widehat{p}_d)'$ is asymptotically unbiased, because

$$\begin{aligned}|E(\widehat{p}_i) - p_i| &= \left|\frac{1}{d}\sum_{h=1}^{d}\left(E\left(\int \widehat{F}_h d\widehat{F}_i\right) - \int F_h dF_i\right)\right| \\ &= \left|\frac{1}{d}\sum_{h=1}^{d}\frac{1}{\lambda_h\lambda_i}\sum_{k=1}^{n}\sum_{\ell=1}^{n}\lambda_{ik}\lambda_{h\ell}\left(E\left(c(X_{ik} - X_{h\ell}\right) - \int F_h dF_i\right)\right| \\ &= \left|\frac{1}{d}\sum_{h=1}^{d}\frac{1}{\lambda_h\lambda_i}\sum_{k=1}^{n}\lambda_{ik}\lambda_{hk}\left(E\left(c(X_{ik} - X_{hk}\right) - \int F_h dF_i\right)\right| \\ &\leq \frac{1}{d}\sum_{h=1}^{d}\frac{1}{\lambda_h\lambda_i}\sum_{k=1}^{n}\left|\lambda_{ik}\lambda_{hk}\left(E\left(c(X_{ik} - X_{hk}\right) - \int F_h dF_i\right)\right| \\ &\leq \frac{1}{d}\sum_{h=1}^{d}\frac{n}{\lambda_h\lambda_i} \leq \frac{1}{d}\sum_{h=1}^{d}\frac{N_0}{\lambda_i} = \frac{N_0}{\lambda_i} \to 0, \lambda_i \to \infty.\end{aligned}$$

2. The strong consistency of $\widehat{\mathbf{p}}$ follows from

$$\begin{aligned}|\widehat{p}_i - p_i| &\leq \frac{1}{d}\sum_{s=1}^{d}\Big|\int \widehat{F}_s d\widehat{F}_i - \int F_s dF_i\Big| \\ &= \frac{1}{d}\sum_{s=1}^{d}\Big|\widehat{F}_s d\widehat{F}_i - \int F_s dF_i - \int F_s d\widehat{F}_i + \int F_s d\widehat{F}_i\Big| \\ &\leq \frac{1}{d}\sum_{s=1}^{d}\Big(\Big|\int (\widehat{F}_s - F_s) d\widehat{F}_i\Big| + \Big|\int F_s d(\widehat{F}_i - F_i)\Big|\Big) \\ &= \frac{1}{d}\sum_{s=1}^{d}\Big(\Big|\int (\widehat{F}_s - F_s) d\widehat{F}_i\Big| + \Big|\int (F_i - \widehat{F}_i) dF_s\Big|\Big) \\ &\leq \frac{1}{d}\sum_{s=1}^{d}\Big(||\widehat{F}_s - F_s||_\infty + ||\widehat{F}_i - F_i||_\infty\Big) \xrightarrow{a.s.} 0,\, \lambda_i, \lambda_s \to \infty.\end{aligned}$$

2.2 Proof of Theorem 1

It is sufficient to prove the theorem only for the i-th component. By adding and subtracting $\int G d\widehat{F}_i$ and $\int G dF_i$, it holds for all $i = 1, \ldots, d$ that

$$\sqrt{n}\int \widehat{G} d(\widehat{F}_i - F_i) = \sqrt{n}\int G d(\widehat{F}_i - F_i) + \sqrt{n}\underbrace{\int (\widehat{G} - G) d(\widehat{F}_i - F_i)}_{Z_i}.$$

If it can be shown, that

$$\sqrt{n}Z_i = \sqrt{n}\int (\widehat{G} - G) d(\widehat{F}_i - F_i) \xrightarrow{p} 0,\, i = 1, \ldots, d,$$

the proof will be complete. It is technically easier to proof the stronger result $E(\sqrt{n}Z_i)^2 \to 0$. Therefore, consider

$$\sqrt{n}Z_i \;=\; \sqrt{n}\int (\widehat{G} - G) d(\widehat{F}_i - F_i) = \frac{1}{d}\sum_{s=1}^{d}\sqrt{n}\int (\widehat{F}_s - F_s) d(\widehat{F}_i - F_i)$$

and thus

$$E\left(\sqrt{n}Z_i\right)^2 \;=\; E\left\{\frac{n}{d^2}\sum_{s=1}^{d}\sum_{t=1}^{d}\int (\widehat{F}_s - F_s) d(\widehat{F}_i - F_i)\int (\widehat{F}_t - F_t) d(\widehat{F}_i - F_i)\right\}.$$

It follows with the same arguments as used by Dobler et al.[7] (see equation (8)), Gao et al.[8] (Lemma 2.1) or Munzel[9] that

$$E\left\{n\int (\widehat{F}_s - F_s) d(\widehat{F}_i - F_i)\int (\widehat{F}_t - F_t) d(\widehat{F}_i - F_i)\right\} \to 0,\; \lambda_i \to \infty,$$

for any fixed pair of (s,t) under **(A1)** and **(A2)**. It remains to show that

$$
\begin{aligned}
& \sqrt{n}\int \widehat{G}d(\widehat{F}_i - F_i) = \sqrt{n}\int Gd(\widehat{F}_i - F_i) + o_p(1) \\
\Leftrightarrow\quad & \sqrt{n}(\widehat{p}_i - p_i) = \sqrt{n}\left(\int Gd\widehat{F}_i - \int F_i d\widehat{G} + 1 - 2p_i\right) + o_p(1) \\
= \quad & \sqrt{n}\left(\frac{1}{\lambda_i}\sum_{k=1}^{n}\lambda_{ik}G(X_{ik}) - \int F_i d\left(\frac{1}{d}\sum_{s=1}^{d}\widehat{F}_s\right) + 1 - 2p_i\right) + o_p(1) \\
= \quad & \sqrt{n}\left(\frac{1}{n}\sum_{k=1}^{n}\frac{n\cdot\lambda_{ik}}{\lambda_i}G(X_{ik}) - \frac{1}{d}\sum_{s=1}^{d}\int F_i d\widehat{F}_s + 1 - 2p_i\right) + o_p(1) \\
= \quad & \sqrt{n}\left(\frac{1}{n}\sum_{k=1}^{n}\frac{n\lambda_{ik}}{\lambda_i}\left(G(X_{ik}) - \frac{1}{d}F_i(X_{ik})\right) - \frac{1}{d}\sum_{s\neq i}\frac{n\lambda_{sk}}{\lambda_s}F_i(X_{sk}) + (1-2p_i)\right) + o_p(1) \\
= \quad & \frac{1}{\sqrt{n}}\sum_{k=1}^{n}(\Psi_{ik} - E(\Psi_{ik})) + o_P(1),
\end{aligned}
$$

which completes the proof.

2.3 Proof of Theorem 2

Note that $\sqrt{n}\mathbf{B}$ is a mean (multiplied by $\sqrt{n}$) of independent random variables

$$
\sqrt{n}\mathbf{B} \quad = \quad \frac{1}{\sqrt{n}}\sum_{k=1}^{n}(\mathbf{\Psi}_k - E(\mathbf{\Psi}_k)), \quad \mathbf{\Psi}_k = (\Psi_{1k},\ldots,\Psi_{dk})'.
$$

Furthermore, note that the random variables $\mathbf{\Psi}_k$ are unformly bounded by Assumption **(A2)** in (**??**). Thus, the multivariate Lindeberg Feller Theorem implies that $\sqrt{n}\mathbf{B}$ is asymptotically normal with covariance matrix $\mathbf{V}_n$ and the result follows from Theorem 1 and Slutzky.

2.4 Proof of Theorem 3

1. Let $\mathbf{y} = (y_1,\ldots,y_d)'$ be a vector of constants. Then it holds

$$
\begin{aligned}
\mathbf{y}'\widehat{\mathbf{V}}_n\mathbf{y} \quad &= \quad \mathbf{y}'\left\{\frac{1}{n-1}\sum_{k=1}^{n}\left(\widehat{\mathbf{\Psi}}_k - \widehat{\boldsymbol{\beta}}_k\right)\left(\widehat{\mathbf{\Psi}}_k - \widehat{\boldsymbol{\beta}}_k\right)'\right\}\mathbf{y} \\
&= \quad \frac{1}{n-1}\sum_{k=1}^{n}\mathbf{y}'\left(\widehat{\mathbf{\Psi}}_k - \widehat{\boldsymbol{\beta}}_k\right)\left(\widehat{\mathbf{\Psi}}_k - \widehat{\boldsymbol{\beta}}_k\right)'\mathbf{y} \\
&= \quad \frac{1}{n-1}\sum_{k=1}^{n}\left(\left(\widehat{\mathbf{\Psi}}_k - \widehat{\boldsymbol{\beta}}_k\mathbf{y}\right)'\right)^2 \geq 0.
\end{aligned}
$$

2. For the diagonal elements, it suffices to show, that

$$\left|\frac{1}{n}\sum_{k=1}^{n}\Big(\big(\widehat{\Psi}_{ik}-\widehat{\beta}_{ik}\big)^2-\big(\Psi_{ik}-E(\Psi_{ik})\big)^2\Big)\right| \xrightarrow{a.s.} 0, n\to\infty, \forall i,$$

since

$$\frac{1}{n-1}\sum_{k=1}^{n}\big(\Psi_{ik}-E(\Psi_{ik})\big)^2=\frac{1}{n}\sum_{k=1}^{n}\big(\Psi_{ik}-E(\Psi_{ik})\big)^2+O\Big(\frac{1}{n}\Big), \forall i.$$

First, we compute the bound of

$$\begin{aligned}|\Psi_{ik}| &= \left|\frac{n\lambda_{ik}}{\lambda_i}\left\{G(X_{ik})-\frac{1}{d}F_i(X_{ik})\right\}-\frac{1}{d}\sum_{s\neq i}\frac{n\lambda_{sk}}{\lambda_s}F_i(X_{sk})\right| \\ &= \left|\frac{1}{d}\underbrace{\sum_{s\neq i}\frac{n\lambda_{ik}}{\lambda_i}}_{\leq dN_0}\underbrace{F_s(X_{ik})}_{\leq 1}-\frac{1}{d}\underbrace{\sum_{s\neq i}\frac{n\lambda_{sk}}{\lambda_s}}_{\leq dN_0}F_i(X_{sk})\right| \leq N_0.\end{aligned}$$

With the same arguments, it follows $|\widehat{\Psi}_{ik}|\leq N_0$, $|\beta_{ik}|\leq N_0$ and $|\widehat{\beta}_{ik}|\leq N_0$.

As $n\to\infty$, it follows that

$$\begin{aligned}&\left|\frac{1}{n}\sum_{k=1}^{n}\Big(\big(\widehat{\Psi}_{ik}-\widehat{\beta}_{ik}\big)^2-\big(\Psi_{ik}-E(\Psi_{ik})\big)^2\Big)\right| \\ &\leq \max_{k=1,\ldots,n}\left|\big(\widehat{\Psi}_{ik}-\widehat{\beta}_{ik}\big)^2-\big(\Psi_{ik}-E(\Psi_{ik})\big)^2\right| \\ &= \max_{k=1,\ldots,n}\left|\big(\widehat{\Psi}_{ik}-\widehat{\beta}_{ik}+\Psi_{ik}-E(\Psi_{ik})\big)\big(\widehat{\Psi}_{ik}-\widehat{\beta}_{ik}-\Psi_{ik}+E(\Psi_{ik})\big)\right| \\ &\leq 4N_0\max_{k=1,\ldots,n}\left|\widehat{\Psi}_{ik}-\widehat{\beta}_{ik}-\Psi_{ik}+E(\Psi_{ik})\right| \\ &\leq 4N_0\max_{k=1,\ldots,n}\Big|\underbrace{\widehat{\Psi}_{ik}-\Psi_{ik}}_{(i)}\Big|+4N_0\max_{k=1,\ldots,n}\Big|\underbrace{\widehat{\beta}_{ik}-E(\Psi_{ik})}_{(ii)}\Big| \xrightarrow{a.s.} 0,\end{aligned}$$

since

$$
\begin{aligned}
(i) &= \Bigg| \frac{n\lambda_{ik}}{\lambda_i}\Big(\widehat{G}(X_{ik}) - \frac{1}{d}\widehat{F}_i(X_{ik})\Big) - \frac{1}{d}\sum_{s\neq i}\frac{n\lambda_{sk}}{\lambda_s}\widehat{F}_i(X_{sk}) \\
&\quad - \left(\frac{n\lambda_{ik}}{\lambda_i}\Big(G(X_{ik}) - \frac{1}{d}F_i(X_{ik})\Big) - \frac{1}{d}\sum_{s\neq i}\frac{n\lambda_{sk}}{\lambda_s}F_i(X_{sk})\right)\Bigg| \\
&\leq \frac{n\lambda_{ik}}{\lambda_i}\Big(|\widehat{G}(X_{ik}) - G(X_{ik})| + \frac{1}{d}|\widehat{F}_i(X_{ik}) - F_i(X_{ik})|\Big) \\
&\quad + \frac{1}{d}\sum_{s\neq i}\frac{n\lambda_{sk}}{\lambda_s}|\widehat{F}_i(X_{sk}) - F_i(X_{sk})| \\
&\leq N_0\Big(||\widehat{G} - G||_\infty + \frac{1}{d}||\widehat{F}_i - F_i||_\infty\Big) + \frac{1}{d}\sum_{s\neq i}N_0||\widehat{F}_i - F_i||_\infty \xrightarrow{a.s.} 0,
\end{aligned}
$$

under assumption **(A2)** and

$$
\begin{aligned}
(ii) &= \Bigg|\left(\frac{n\lambda_{ik}}{\lambda_i}\Big(p_i - \frac{1}{d}p^{(ii)}\Big) - \frac{1}{d}\sum_{s\neq i}\frac{n\lambda_{sk}}{\lambda_s}p^{(is)}\right) \\
&\quad - \left(\frac{n\lambda_{ik}}{\lambda_i}\Big(\widehat{p}_i - \frac{1}{d}\widehat{p}^{(ii)}\Big) - \frac{1}{d}\sum_{s\neq i}\frac{n\lambda_{sk}}{\lambda_s}\widehat{p}^{(is)}\right)\Bigg| \\
&\leq N_0\Big(|\widehat{p}_i - p_i| + \frac{1}{d}|\widehat{p}^{(ii)} - p^{(ii)}|\Big) + \frac{1}{d}\sum_{s\neq i}N_0|\widehat{p}^{(is)} - p^{(is)}| \xrightarrow{a.s.} 0.
\end{aligned}
$$

For the off diagonal elements, it holds that

$$
\begin{aligned}
&\frac{1}{n}\sum_{k=1}^{n}\underbrace{\Big(\big(\widehat{\Psi}_{ik} - \widehat{\beta}_{ik}\big)\big(\widehat{\Psi}_{jk} - \widehat{\beta}_{jk}\big) - \big(\Psi_{ik} - E(\Psi_{ik})\big)\big(\Psi_{jk} - E(\Psi_{jk})\big)\Big)}_{\Delta_{ik}} \\
&\leq \max_{k=1,\dots,n}\Big|\Delta_{ik}\Big| \xrightarrow{a.s.} 0,
\end{aligned}
$$

since

$$
\begin{aligned}
|\Delta_{ik}| &= \Big|\big(\widehat{\Psi}_{ik}-\widehat{\beta}_{ik}\big)\big(\widehat{\Psi}_{jk}-\widehat{\beta}_{jk}\big)-\big(\Psi_{ik}-E(\Psi_{ik})\big)\big(\Psi_{jk}-E(\Psi_{jk})\big)\Big| \\
&= \Big|\big(\widehat{\Psi}_{ik}-\widehat{\beta}_{ik}\big)\big(\widehat{\Psi}_{jk}-\widehat{\beta}_{jk}\big)-\big(\Psi_{ik}-E(\Psi_{ik})\big)\big(\Psi_{jk}-E(\Psi_{jk})\big) \\
&+\big(\widehat{\Psi}_{ik}-\widehat{\beta}_{ik}\big)\big(\Psi_{jk}-E(\Psi_{jk})\big)-\big(\widehat{\Psi}_{ik}-\widehat{\beta}_{ik}\big)\big(\Psi_{jk}-E(\Psi_{jk})\big)\Big| \\
&= \Big|\big(\widehat{\Psi}_{ik}-\widehat{\beta}_{ik}\big)\Big[\big(\widehat{\Psi}_{jk}-\widehat{\beta}_{jk}\big)-\big(\Psi_{jk}-E(\Psi_{jk})\big)\Big] \\
&+\big(\Psi_{jk}-E(\Psi_{jk})\big)\Big[\big(\widehat{\Psi}_{ik}-\widehat{\beta}_{ik}\big)-\big(\Psi_{ik}-E(\Psi_{ik})\big)\Big]\Big| \\
&\leq \Big|\big(\widehat{\Psi}_{ik}-\widehat{\beta}_{ik}\big)\Big[\big(\widehat{\Psi}_{jk}-\widehat{\beta}_{jk}\big)-\big(\Psi_{jk}-E(\Psi_{jk})\big)\Big]\Big| \\
&+\Big|\big(\Psi_{jk}-E(\Psi_{jk})\big)\Big[\big(\widehat{\Psi}_{ik}-\widehat{\beta}_{ik}\big)-\big(\Psi_{ik}-E(\Psi_{ik})\big)\Big]\Big| \\
&\leq 2N_0\Big[\big(\widehat{\Psi}_{jk}-\widehat{\beta}_{jk}\big)-\big(\Psi_{jk}-E(\Psi_{jk})\big)\Big] \\
&+2N_0\Big[\big(\widehat{\Psi}_{ik}-\widehat{\beta}_{ik}\big)-\big(\Psi_{ik}-E(\Psi_{ik})\big)\Big] \\
&\leq 2N_0\left[\Big|\widehat{\Psi}_{jk}-\Psi_{jk}\Big|+\Big|\widehat{\beta}_{jk}-E(\Psi_{jk})\Big|+\Big|\widehat{\Psi}_{ik}-\Psi_{ik}\Big|+\Big|\widehat{\beta}_{ik}-E(\Psi_{ik})\Big|\right] \\
&\xrightarrow{a.s.} 0.
\end{aligned}
$$

References

1. Domhof S, Brunner E and Osgood DW. Rank procedures for repeated measures with missing values. *Sociological Methods & Research* 2002; 30(3): 367–393.
2. Santos MS, Pereira RC, Costa AF et al. Generating synthetic missing data: A review by missing mechanism. *IEEE Access* 2019; 7: 11651–11667.
3. Amro L, Konietschke F and Pauly M. Incompletely observed nonparametric factorial designs with repeated measurements: A wild bootstrap approach, 2021.
4. Zhu B, He C and Liatsis P. A robust missing value imputation method for noisy data. *Appl Intell* 2012; 36: 61–74.
5. R Core Team. *R: A Language and Environment for Statistical Computing*. R Foundation for Statistical Computing, Vienna, Austria, 2013. URL http://www.R-project.org/.
6. Domhof S. *Nichtparametrische relative Effekte (Doctoral dissertation, Niedersächsische Staats-und Universitätsbibliothek Göttingen)*. 2001. URL https://ediss.uni-goettingen.de/bitstream/handle/11858/00-1735-0000-000D-F284-4/domhof.pdf?sequence=1.
7. Dobler D, Friedrich S and Pauly M. Nonparametric manova in meaningful effects. *Annals of the Institute of Statistical Mathematics* 2019; 72: 1–26.
8. Gao X, Alvo M, Chen J et al. Nonparametric multiple comparison procedures for unbalanced one-way factorial designs. *Journal of Statistical Planning and Inference* 2008; 138(8): 2574 – 2591.

9. Munzel U. Nonparametric methods for paired samples. *Statistica Neerlandica* 1999; 53(3): 277–286.
