# Supplementary material for: Ranking procedures for repeated measures designs with missing data: Estimation, testing and asymptotic theory
Source: Stat Methods Med Res. 2021 Nov 29;31(1):105–18. doi: 10.1177/09622802211046389 (PMC8721540; doi:10.1177/09622802211046389)
Supplement: sj-pdf-1-smm-10.1177_09622802211046389 - Supplemental material for Ranking procedures for repeated measures designs with missing data: Estimation, testing and asymptotic theory [file sj-pdf-1-smm-10.1177_09622802211046389.pdf]

Point-By-Point Response to "Ranking Procedures for Repeated Measures Designs with Missing Data - Estimation, Testing and Asymptotic Theory"

June 23, 2021

First, we would like to thank the Editor, the Associate Editor and the two anonymous referees for their careful reading of our manuscript. The comments are very helpful and improve our paper. We highlighted all changes in blue for readability.

1 Reviewer 1

1. In the paper, the authors claim that the method is suitable for both MCAR and MAR missing mechanisms. In the simulation, both scenarios are simulated. It would be helpful to readers if the authors can provide some justifications on why the method is applicable to both missing mechanisms.
   Response: Many thanks for your comment. We have added a short statement, indicating that we compared MCAR and MAR scenarios with overall similar missing rates and saw no differences between the type-I error rates in MCAR and MAR scenarios. Although our theory is developed under the MCAR assumption, we therefore conclude that our method seems to be also applicable in MAR scenarios.

2 Reviewer 2

1. (equation 6) N_0 was never defined. Is N_0 an arbitrary constant? If so, please state so.
   Response: Many thanks for noting it. N_0 is indeed an arbitrary constant. We changed it accordingly.

2. (p. 9 first lines) You state: "Note that each vector $\boldsymbol{\Psi}_k = (\Psi_{1k}, ..., \Psi_{dk})'$ is centered with its own specific expectation and therefore $\widetilde{\boldsymbol{V}}_N$ is not the empirical covariance matrix of the vectors $\boldsymbol{\Psi}_k$." For some reason I needed to read this sentence several times to understand it. I think it would have helped me if you had added the phrase "in the definition of $\widetilde{\boldsymbol{V}}_N$" after "Note that".
   Response: Many thanks for your valuable comment. We added the phrase in the manuscript.

3. (p. 11, lines 16 and 28) You use the phrase "multiple level α" and then referenced Bretz, et al 2001 [ref. 19]. I am unfamiliar with the term and searched that paper, and it did not use the phrase either. Do you mean that if you reject each $H_0^{(l)}$ when $|T_\ell| \geq z_{1-\alpha,2,\boldsymbol{R}}$ for $\ell = 1, ..., q$ then that retains the familywise type I error rate at α? I am also unfamiliar with the phrase "two-sided $(1-\alpha)$-equicoordinate quantile of the $N(0, \boldsymbol{R})$ distribution", which is also not in ref. 19 (I searched for "equicoordinate" and it was not in the paper). Could you explain that phrase another way? Or give a specific page number for the definition in ref. 19.
   Response: Yes, you are right. We mean with "multiple α level" that the familywise type-I error rate is controlled and added a short phrase to the manuscript.
   The two-sided $(1-\alpha)$-equicoordinate quantile satisfies the condition $P(|T_1| < z_{1-\alpha,2,\boldsymbol{R}}, ..., |T_q| < z_{1-\alpha,2,\boldsymbol{R}}) = 1-\alpha$ for $(T_1, ..., T_q)' \sim N(\boldsymbol{0}, \boldsymbol{R})$. We also added this short explanation to the manuscript. Bretz et al. (2001) show how to calculate this equicoordinate quantile, whereas Konietschke et al. (2012) gave this specific quantile the name "equicoordinate quantile". We therefore changed the citation from Bretz et al. to Konietschke et al. and added that Bretz et al. demonstrated how to obtain the quantile numerically. We hope it is sufficiently clear now.

4. (p. 16, line 32) typo: "(??)".

Response: Many thanks for noting it. We corrected it in the supplement.

3 Other changes

We detected a missing 'hat' at page 11 in the formula of the consistent estimator $\widehat{\boldsymbol{R}}_n$, we corrected that accordingly. Furthermore, we corrected typos and a beginning sentence of Section 6 for readability.
